# Vibrationally Mediated Dzyaloshinskii-Moriya Interaction as the Origin of Chirality-Induced Spin Selectivity in Donor–Acceptor Molecules

**DOI:** 10.1021/acs.nanolett.6c01653

**Published:** 2026-07-14

**Authors:** Alessandro Chiesa, D. K. Andrea Phan Huu, Arianna Cantarella, Leonardo Celada, Michael R. Wasielewski, Paolo Santini, Stefano Carretta

**Affiliations:** † Dipartimento di Scienze Matematiche, Fisiche e Informatiche, 9370 Università di Parma, Parco Area delle Scienze, 53/A, I-43124 Parma, Italy; ‡ Gruppo Collegato di Parma, INFN-Sezione Milano-Bicocca, I-43124 Parma, Italy; § UdR Parma, INSTM, I-43124 Parma, Italy; ∥ Department of Chemistry, Institute for Quantum Information Research and Engineering, and Center for Molecular Quantum Transduction, 3270 Northwestern University, Evanston, Illinois 60208-3113, United States

**Keywords:** Chirality-Induced Spin-Selectivity, Dzyaloshinskii-Moriya interaction, Spin Polarization, Vibrations

## Abstract

Chirality-induced spin selectivity (CISS) was recently observed in photoexcited donor-chiral bridge-acceptor molecules, but a predictive theory able to explain available experiments is still lacking. Here, we show that torsional modes modulating hopping and spin–orbit coupling give rise to a Dzyaloshinskii-Moriya interaction between the transferred electron and the one sitting on the donor, producing high spin polarization for realistic parameters. Our model introduces a low-energy scale in the spin dynamics that explains the magnetic field dependence observed in EPR measurements and predicts a nontrivial temperature dependence, as demonstrated by numerical simulations. The present theory lays the foundations for future test bed experiments and for the design of applications in spintronics and quantum technologies.

## Graphical abstract

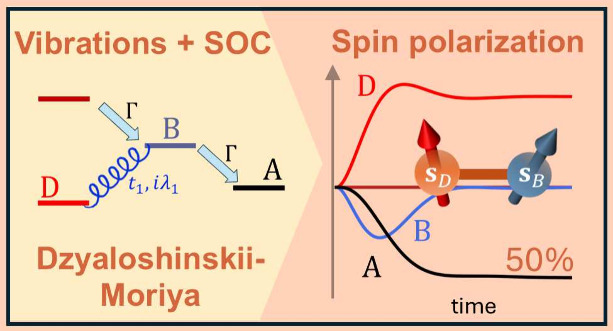

After many different observations in photoemission, transport, and polarization-on-surface experiments,^1–5^ Chirality-Induced Spin Selectivity (CISS) was recently evidenced also in donor–acceptor molecules with a chiral bridge (*D* – χ – *A*) in solution.^6–8^ In these systems, photoinduced electron transfer (ET) produces a sizable triplet component in the radical-pair state, observed by time-resolved electron paramagnetic resonance (EPR) and completely absent in achiral analogs.

The important simplification of the experimental setup in ET, replacing complex interfaces with a single donor spin, can be the key for a substantial step forward in the comprehension of the phenomenon.^9,10^ The fundamental dilemma is how to reconcile the small spin–orbit coupling of organic chiral molecules with the large electronic energy gaps between the states involved in the electron motion.^11^ This prevents single-electron models to yield a significant spin polarization,^12,13^ unless very strong spin relaxation is assumed.^14,15^ The transferred electron must therefore be involved in some form of interaction.^16^ For instance, spin polarization was shown to arise from its coupling with the electron remaining on the donor, combined with SOC.^17,18^ Significant polarization was also demonstrated in a many-electron picture of the chiral bridge, by including electron–electron correlations^19,20^ or by coupling electrons with low-energy vibrations.^21–28^

However, molecular systems undergoing ET typically show large energy gaps between completely filled (HOMO) and empty (LUMO) bridge orbitals, making electron–electron interactions ineffective. An example is provided by the PXX-NMI_2_–NDI molecule,^6^ on which we recently performed an extensive *ab initio* study.^29^ Nonetheless, experimental evidence of a strong magnetic-field dependence of CISS^8^ implies the existence of small energy gaps involved in the effect.

We focus on Peierls vibrations modulating both hopping and SOC,^30–34^ such as torsional modes typical of chiral molecules,^29,35^ and show they can give rise to an effective spin–spin interaction between the moving electron and the one on the donor of the same form found for ET in ref 18, but potentially much larger. In particular, a Dzyaloshinskii-Moriya interaction (DMI) of the same order of the isotropic exchange arises. By mixing singlet and triplet during ET, this DMI produces a large spin polarization and a sizable triplet component in the charge-separated radical pair, perfectly compatible with experiments.

A similar mechanism can also arise at metal–organic interfaces between localized surface spins and the electron traveling through the chiral molecule^36,37^ and in a many-body description of a chiral bridge^22,28^ for which we derive here a vibrationally mediated electron–electron coupling. Hence, the present results contribute to understanding CISS also in transport.

We demonstrate the key role of the vibrationally mediated DMI for CISS by numerically solving the ET dynamics in a Redfield framework. We study the dependence of the spin polarization on model parameters, finding large values in a realistic range. Our model explains the observed magnetic-field dependence of the triplet component probed by EPR experiments. In spite of the small energy scale introduced by spin–spin interactions, the effect is robust in temperature and shows a nontrivial temperature dependence that could be tested in future experiments.

The sizable spin polarization we predict (not limited to 50% for nontrivial models) will be the starting point to design applications in quantum technologies.

## Effective Spin Hamiltonian

Photoinduced ET in molecules displaying CISS^6–8^ can be described by a sequential incoherent hopping from the excited donor orbital (De) to the acceptor (A), via an intermediate bridge orbital (B) as sketched in Figure 1-(a). During the process, the transferred electron interacts with the one remaining on the donor (D) HOMO. Such interaction arises both from a delocalization of the electronic wave function through hopping and SOC and from the corresponding modulations induced by vibrations. The related dynamics following photoexcitation can be described by incoherent spin-independent transfer rates Γ from De to B to A, combined with a coherent evolution ruled by the Hamiltonian *H* = *H*_0_ + *H*_1_

1 $$H_{0}=\Delta \underset{\sigma =↑,↓}{\sum }c_{B\sigma }^{†}c_{B\sigma }+U\underset{j=D,B}{\sum }n_{j↑}n_{j↓}+\underset{\nu }{\sum }\hbar \omega_{\nu }a_{\nu }^{†}a_{\nu }$$

$$H_{1}=\left(t+i\lambda \right)c_{B↑}^{†}c_{D↑}+\left(t-i\lambda \right)c_{B↓}^{†}c_{D↓}+\underset{\nu }{\sum }\left(a_{\nu }+a_{\nu }^{†}\right)\left[\left(t_{1\nu }+i\lambda_{1\nu }\right)c_{B↑}^{†}c_{D↑}+\left(t_{1\nu }-i\lambda_{1\nu }\right)c_{B↓}^{†}c_{D↓}\right]+h.c.$$ 2

where *c*_*jσ*_^†^ (*c*_*jσ*_) are Fermionic creation (annihilation) operators of an electron with spin σ either on the donor HOMO or on the bridge LUMO (*j* = D, B) and *n*_*jσ*_ = *c*_*jσ*_^†^ *c*_*jσ*_; *a*_ν_^†^ (*a*_ν_) is a bosonic creation (annihilation) operator of a mode of frequency ℏω_*ν*_. The Hamiltonian is partitioned to separate the leading terms diagonal in the occupation number basis (the energy gap Δ, the on-site Coulomb repulsion *U*, and the vibration energy) from the weaker perturbative contributions in *H*_1_. This term accounts for the spin independent hopping (of strength *t*) and SOC (parametrized by λ) as well as for their coupling to the mode ν, with respective strengths *t*_1*ν*_ and λ_1*ν*_. [The electronic system could also be coupled to Holstein modes modulating on-site energies, but these cannot mediate a spin–spin interaction and hence are not considered here.] For simplicity, the static and vibrationally modulated SOC are assumed axial and originate from the molecular chirality.^17,18,27^

**Figure 1:**
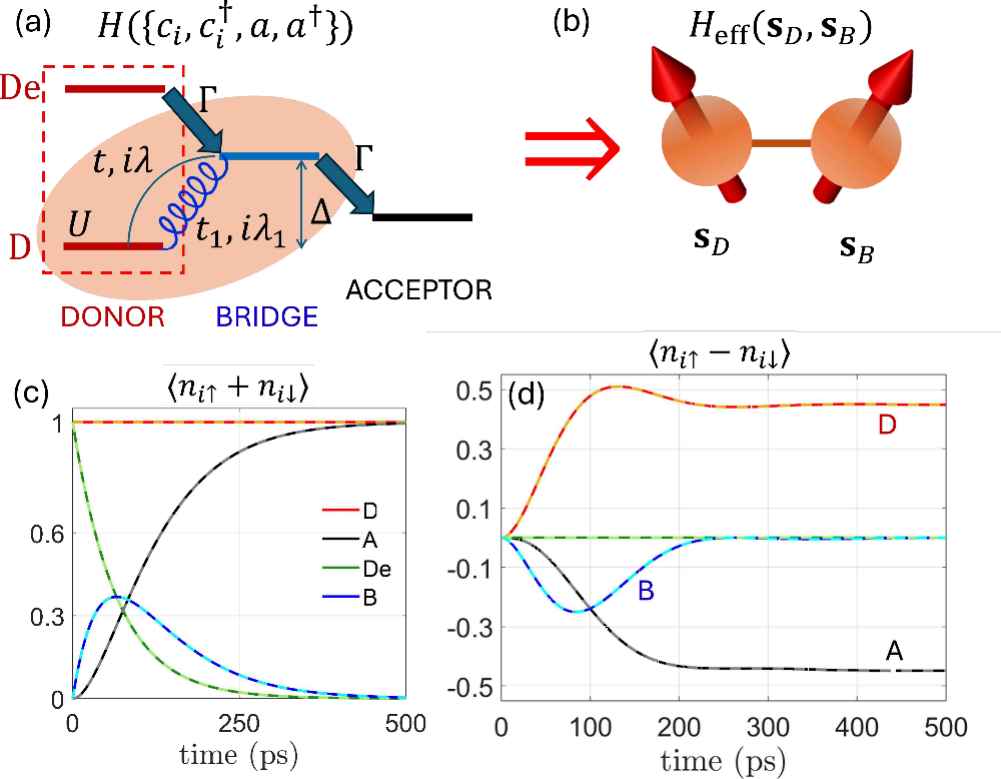
(a) Scheme of the minimal electron-transfer model with HOMO and LUMO on the donor (D, De), an empty intermediate orbital on the bridge (B) and one on the acceptor (A). The dynamics is ruled by incoherent spin-independent jumps from De to B and from B to A at rates Γ and by a Hamiltonian *H* including Fermionic and bosonic degrees of freedom. (b) The Hamiltonian *H* with a single electron on *D* and one on *B* is mapped onto an effective spin Hamiltonian involving spin operators **s**_*D*_ and **s**_*B*_. (c,d) Simulated time evolution of the charge ⟨*n*_*i*↑_ + *n*_*i*↓_⟩ (c) and of the local spin polarization 2⟨*s*_*zi*_⟩ = ⟨*n*_*i*↑_ – *n*_*i*↓_⟩ (d) on different orbitals. Solid lines: simulation with the full Hamiltonian *H* and up to 6 bosons. Dashed lines: simulation with the effective Hamiltonian *H*_eff_. Parameters: *t* = 1 meV, λ = 0.1 meV, *U* = 3.5 eV, Δ = 5 eV, *t*_1_ = 1 meV, λ_1_ = 1 meV, ℏω = 2 meV, *J*_*CE*_ = −10^–3^ meV, Γ = 5 × 10^–3^ meV and we initialized the system with a fixed number of bosons *n* = 3.

Since *t*, λ, *t*_1ν_, λ_1ν_ ≪ *U*, Δ, charge is practically localized on D and B in the intermediate ET step, and hence we can consider *H*_1_ acting as a second-order perturbation on *spin-states*

$$\left|\sigma \sigma^{\prime }\rangle_{\left\{n_{\nu }\right\}}\equiv c_{D\sigma }^{†}c_{B\sigma \prime }^{†}\underset{\nu }{\prod }\frac{{\left(a^{†}\right)}^{n_{\nu }}}{\sqrt{n_{\nu }!}}\right|⌀\rangle ,,\sigma ,\sigma^{\prime }=↑,↓$$ 3

where *n*_*v*_ is the number of bosons in mode ν, and the bosons state is factorized from the electronic one. This treatment results in an effective spin Hamiltonian of the form

4 $$H_{\mathrm{eff}}=Js_{D}\cdot s_{B}+J_{D}\left(2s_{zD}s_{zB}-s_{xD}s_{xB}-s_{yD}s_{yB}\right)+D_{z}\left(s_{xD}s_{yB}-s_{yD}s_{xB}\right)$$

where the three contributions account for an isotropic, axial anisotropic, and antisymmetric (Dzyaloshinskii-Moriya) exchange (see^38^ and the Supporting Information). The values of the couplings are

5a $$J=J_{CE}+\frac{2t^{2}-2\lambda^{2}/3}{\Delta^{\prime }}+2\underset{\nu }{\sum }\left(t_{1\nu }^{2}-\frac{\lambda_{1\nu }^{2}}{3}\right)f\left(n_{\nu }\right)$$

5b $$J_{D}=\frac{4\lambda^{2}}{3\Delta^{\prime }}+\underset{\nu }{\sum }\frac{4\lambda_{1\nu }^{2}}{3}f\left(n_{\nu }\right)$$

5c $$D_{z}=-\frac{4\lambda t}{\Delta^{\prime }}-\underset{\nu }{\sum }4\lambda_{1\nu }t_{1\nu }f\left(n_{\nu }\right)$$

with 1/Δ′ = 1/(*U* – Δ) + 1/(*U* + Δ) and

$$f\left(n_{\nu }\right)=\frac{n_{\nu }+1}{U-\Delta +\hbar \omega_{\nu }}+\frac{n_{\nu }+1}{U+\Delta +\hbar \omega_{\nu }}+\frac{n_{\nu }}{U-\Delta -\hbar \omega_{\nu }}+\frac{n_{\nu }}{U+\Delta -\hbar \omega_{\nu }}\approx \frac{2n_{\nu }+1}{\Delta^{\prime }}$$ 6

The last approximation holds because ℏω_ν_ ≪ *U*, Δ ∼ several eV. Note that we consider here low-frequency torsional modes (typically in the ∼1–10 meV range), but the precise value of the vibrational frequency does not qualitatively alter our results. It only renormalizes the effective couplings in eq 5 through the value of *n*_*ν*_. Besides the second-order contributions, eq 5a includes a first-order direct exchange interaction, *J*_*CE*_.

As already evidenced in ref 18, the three terms in eq 4 (including the DMI) also emerge in the absence of vibrational coupling (*t*_1ν_ = λ_1ν_ = 0). Nonetheless, the couplings are significantly amplified by vibrations. Indeed, (i) different modes may provide additive contributions; (ii) the factor *f*(*n*_ν_) gives an enhancement which becomes increasingly important with temperature; and (iii) we can expect *t*_1ν_ and λ_1ν_ of the same order and often larger than *t* and λ.^29,31,32,39,40^ Specifically, in π-conjugated systems, due to high torsional flexibility, ratios of λ_1ν_/λ ∼ 5–10 have been reported for some modes.^41^ We also explore values of *t*_1ν_ comparable to *t* (as found in ref 29, where however both *t* and *t*_1ν_ were significantly larger for a specific mode).

Since *U* < Δ (as required to get a stable state before photoexcitation) and *t* > λ, the static contribution to *J* is ferromagnetic (negative), as *J*_*CE*_.

Only axial components appear in Hamiltonian ([Disp-formula eq4]), because we started for simplicity from an axially symmetric Hamiltonian ([Disp-formula eq2]). A different form of the SOC would lead to other components of the DMI in *H*_eff_, but this would not qualitatively alter our conclusions.

Finally, it is worth noting that second-order perturbation theory provides a very good approximation, even for *t*_1ν_, λ_1ν_ > ℏω, because *t*_1ν_, λ_1ν_ ≪ *U* – Δ, i.e. the exact eigenstates of *H* are very close to the factorized states in eq 3 (see Supporting Information). Hence, we can trace out the vibrations and study the dynamics ruled by *H*_eff_.

## Large Spin Polarization

We simulate the ET dynamics by numerically integrating the Redfield equation with Hamiltonian *H*_0_ + *H*_eff_ and spin-independent jump operators √Γ∑_σ_ *c*_*Bσ*_^†^ *c*_*Deσ*_ and √Γ∑_σ_ *c*_*Aσ*_^†^ *c*_*Bσ*_. We consider an effective Born-Markov master equation (S12), commonly adopted for incoherent electron transfer,^42,43^ where we assumed no frequency dependence of the bath spectral density over the relevant energy range and unidirectional electron transfer. We have checked that the inclusion of a spin-dependent ET rate does not practically affect our results (see the Supporting Information). For computational reasons we include a single effective boson mode (thus removing the pedices ν), keeping in mind that in real systems the couplings can be enhanced by the sum on several modes in eq 5. An example of the computed time evolution of charge and spin polarization along the chiral axis is reported in Figure 1-(c,d) considering a vibrational mode initialized in the excited state with quantum number *n* = 3. Results obtained by truncating to 6 vibrational basis states are practically superimposed to the perturbative treatment (dashed vs solid lines), demonstrating the validity of the effective spin Hamiltonian (whose derivation is based on adding/removing only a single vibrational quantum).

The origin of the spin polarization is the vibrationally mediated DMI, which mixes singlet (|*S*⟩) and triplet (|*T*_0_⟩,|*T*_+_⟩,|*T*_–_⟩). In the factorized basis, these states correspond to |*S*⟩ = (|↑↓⟩ – |↓↑⟩)/√2, |*T*_0_⟩ = (|↑↓⟩ + |↓↑⟩)/√2, |*T*_+_⟩ = |↑↑⟩, |*T*_–_⟩ = |↓↓⟩. Starting from a singlet, this in general yields a triplet component and both a real and an imaginary coherence in the charge-separated S-T basis. Therefore, we study the behavior of three different observables, namely the spin polarization (*P*_*z*_), the imaginary singlet–triplet coherence (*C*_*i*_), and the triplet component (*P*_*T*_). In the present axial model, these are given by

7a $$P_{z}=s_{zD}-s_{zA}=\left|S\right\rangle \left\langle T_{0}\right|+\left|T_{0}\right\rangle \left\langle S\right|$$

$$C_{i}=s_{xD}s_{yA}-s_{yD}s_{xA}=i\left(\left|S\right\rangle \langle T_{0}|-\left|T_{0}\right\rangle \langle S|\right)/2$$ 7b

7c $$P_{T}=3/4+s_{D}\cdot s_{A}=\left|T_{0}\right\rangle \left\langle T_{0}\right|$$

Remarkably, for realistic parameters, spin polarization accumulates on A, because it undergoes coherent oscillations with angular frequency $\sqrt{{\left(J+J_{D}\right)}^{2}+D_{z}^{2}}$ , which is comparable to the ET rates. The restriction of the relevant dynamics to the spin subspace (in which *J* and *D*_*z*_ are comparable) is the key to achieving high values of *P*_*T*_, *P*_*z*_, and *C*_*i*_.

To provide a realistic description of molecules displaying CISS (without restricting to a specific one), we derive the parameters *U*, Δ, *t*, and λ from ab initio calculations^44–46^ on PXX-NMI_2_–NDI (see the Supporting Information and the caption of Figure 2), and we perform numerical simulations as a function of *t*_1_ and λ_1_. [Since the orientation of the spin–orbit coupling vector depends on the details of the molecular structure, we consider for λ in our minimal axial model the magnitude of the spin–orbit coupling vector, as shown in the Supporting Information.] We set Γ = 5 × 10^–3^ meV, corresponding to a time constant for ET ℏ/Γ ≈ 100 ps, so that ET completes in a few hundred ps. Unlike the benchmark simulations reported in Figure 1, hereafter we consider a thermal equilibrium state of the vibrational mode at different temperatures.

**Figure 2:**
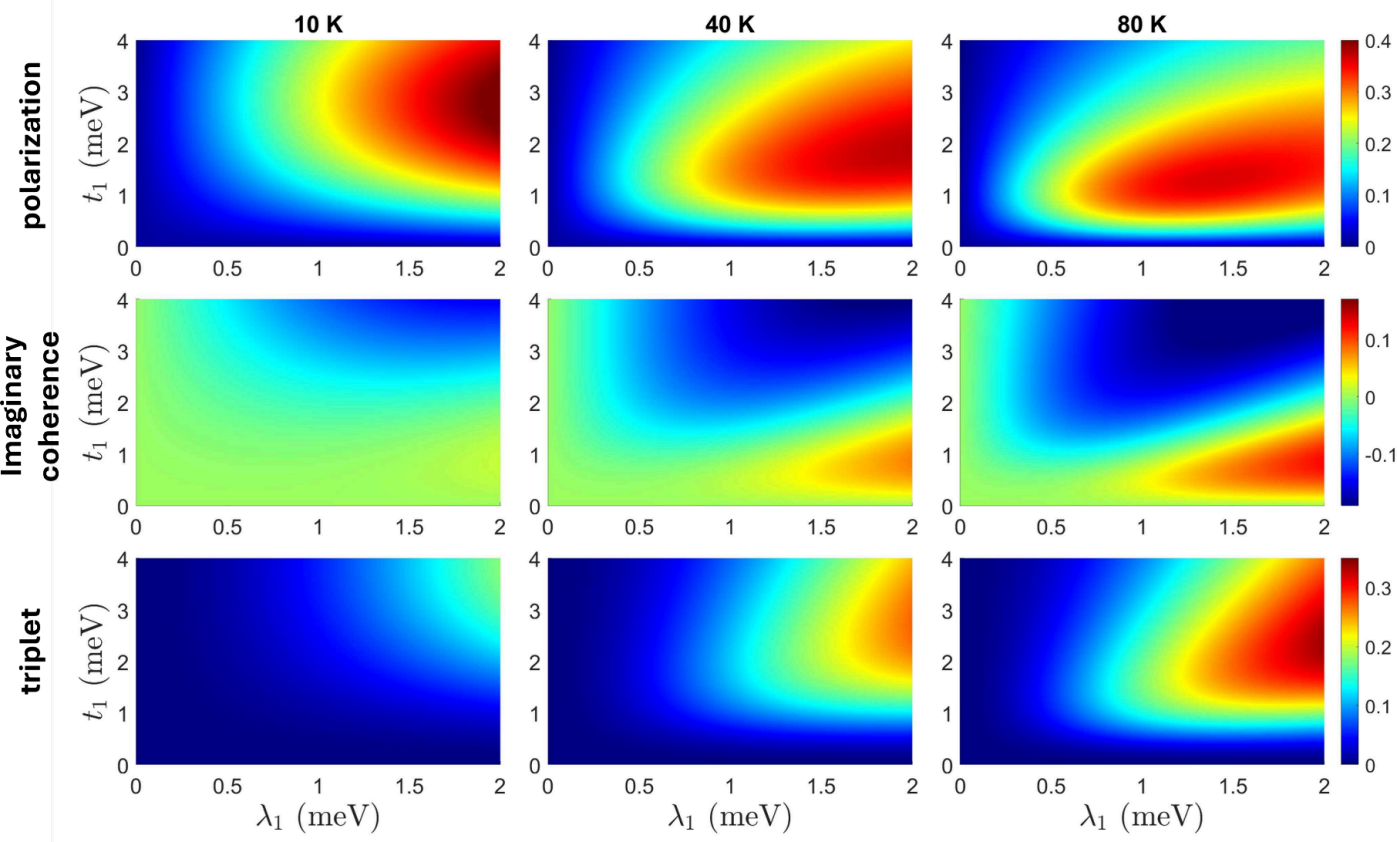
Top panels: spin polarization ⟨*P*_*z*_⟩, corresponding to twice the real part of the singlet–triplet coherence. Middle panels: imaginary component of the singlet–triplet coherence ⟨*C*_*i*_⟩. Bottom panels: triplet population ⟨*P*_*T*_⟩. All the values are at the end of the ET, as a function of λ_1_ and *t*_1_. Other parameters of the simulation: *t* = 1 meV, Γ = 5 × 10^–3^ meV (corresponding to ET time in the few hundreds of ps range ℏ/Γ ≈ 100 ps), direct exchange contribution *J*_*CE*_ = −10^–3^ meV, λ = 0.1 meV, *U* = 3.5 eV, Δ = 5 eV, ℏω_0_ = 2 meV. Simulations with larger *t*_1_ and Γ are reported in the Supporting Information.

Results for the accumulated spin polarization, imaginary coherence, and triplet component as a function of *t*_1_ and λ_1_ are reported in Figure 2. We start our analysis from zero magnetic field and by exploring a similar range of values for *t*_1_ and λ_1_, but we anticipate that in applied field large singlet–triplet mixing and polarization can be also obtained for smaller λ_1_/*t*_1_.

The first line of Figure 2 shows the expectation value of *P*_*z*_ at the end of ET, at different temperatures. By raising the temperature, more vibrational quanta are introduced, hence the values of *J*, *J*_*D*_, and *D*_*z*_ increase. This leads to a corresponding growth in the oscillation frequency, which, for a fixed ET time, shifts both the maximum value of ⟨*P*_*z*_⟩ and its position in {λ_1_, *t*_1_}. While at lower temperatures the ET time matches the maximum of the first oscillation in ⟨*P*_*z*_⟩ for λ_1_ ≈ *t*_1_ ≈ 2 meV, at higher temperatures the value of ⟨*P*_*z*_⟩ is already decreasing before the end of the ET. By reducing the ET time, the maximum of ⟨*P*_*z*_⟩ is reached at higher temperatures (see the Supporting Information). Remarkably, ⟨*P*_*z*_⟩ is robust with temperature despite the small energy scales of *H*_eff_, because the initial photoexcited state is out-of-equilibrium and the vibrational mode ν remains at thermal equilibrium for the whole dynamics. [Higher energy modes driving ET can be out of thermal equilibrium, but here they only contribute to the rates Γ.]

In the second line of Figure 2 we show ⟨*C*_*i*_⟩. Its maximum grows with temperature, and it changes sign when *J* does, i.e. when *t*_1_ dominates over λ_1_.

The last line reports the final triplet component ⟨*P*_*T*_⟩, which increases with λ_1_ and has a maximum in *t*_1_, since the singlet–triplet mixing decreases at larger *t*_1_.

A few remarks are now in order. First, the frequency of the vibrational modes affects the computed observables only indirectly, by renormalizing the effective values of *D*_*z*_ and *J*, without altering their ratio. This implies that large singlet–triplet mixing can be obtained even for higher frequency modes but at longer times. This, in turn, will change the temperature dependence of the observables if we keep Γ fixed.

Another interesting point concerns the damping of vibrational modes, which is expected in a condensed phase environment. In the present perturbative regime, vibrations are only *virtually* excited, so damping affects only a small fraction ∼ *t*_1_/Δ′ (or λ_1_/Δ′) of the wave function, inducing its relaxation to the doubly occupied donor HOMO. Consequently, the main effect on the spin observables (starting from a thermal vibrational state) is a renormalization of damping by a factor ∼ *t*_1_/Δ′ ∼ 10^–3^. We have verified this by including vibrational damping in the Redfield equation for the open system described by *H*_0_ + *H*_1_. Indeed, the spin polarization remains practically unaltered even in an overdamped regime with vibrational loss of 10–100 meV, significantly larger than ℏω_*ν*_ (see the Supporting Information), thus confirming the soundness of our model. The same conclusion holds for the triplet component ⟨*P*_*T*_⟩.

## Comparison with Experiments

The triplet component of the radical pair is the quantity accessed by time-resolved electron paramagnetic resonance (TREPR) experiments, the technique used so far to probe CISS in photoinduced ET.^9,10,47,48^ By focusing on the low-energy spin dynamics, the present theory can reproduce experimental observations and, in particular, the nontrivial magnetic field dependence.

To this aim, we include in the spin Hamiltonian a Zeeman term of the form

8 $$H_{\mathrm{Zeem}}=\mu_{B}\underset{i}{\sum }g_{i}B\cdot s_{i}$$

where μ_*B*_ is the Bohr magneton, *g*_*i*_ is the *g*-factor of site *i*, and **B** is the magnetic field. We use *g*_*D*_ = 2.0023, *g*_*B*_ = 2.003, and *g*_*A*_ = 2.0038, but the precise values are not relevant for our results. We consider **B** perpendicular to the chiral axis, because this orientation is the most sensitive to CISS in experiments performed on isotropic solutions or on molecules in liquid crystals (as done so far). Liquid crystals orient the direction of chiral molecules but leave the two orientations equally likely. Therefore, we perform simulations on ensembles including both orientations of the molecules but fixed direction. Since the Hamiltonian is no longer axial, DMI will in general mix the initial singlet also with |*T*_+_⟩ and |*T*_–_⟩, where the quantization axis is given by the field orientation. Hence, the triplet population becomes *P*_*T*_ = |*T*_0_⟩ ⟨*T*_0_| + |*T*_+_⟩ ⟨*T*_+_| + |*T*_–_⟩ ⟨*T*_–_|.

To understand the magnetic field dependence we plot in Figure 3-(a) the energy level diagram of the two-spin system as a function of *B* for a fixed number of bosons *n* = 0 (continuous lines) or *n* = 1 (dashed). We remind that in the present perturbative regime spin eigenstates are only slightly mixed with vibrations and the dynamics is ruled by the effective spin Hamiltonian ([Disp-formula eq4]). At zero field triplet levels are practically degenerate (*J*_*D*_ is small) and are split by the isotropic exchange *J* from the higher energy singlet. Then, we note an avoided level crossing (AC) at 0.7 and 2.1 T for the solid and dashed lines, between |*S*⟩ and the *M* = 1 component of the triplet |*T*_+_⟩ along the field. The width of the AC is determined by *D*_*z*_ and hence increases with *n*. [In the present minimal axial model we obtain an AC for any θ ≠ 0. For nonaxial SOC and hence DMI one could get ACs also for θ = 0.]

**Figure 3:**
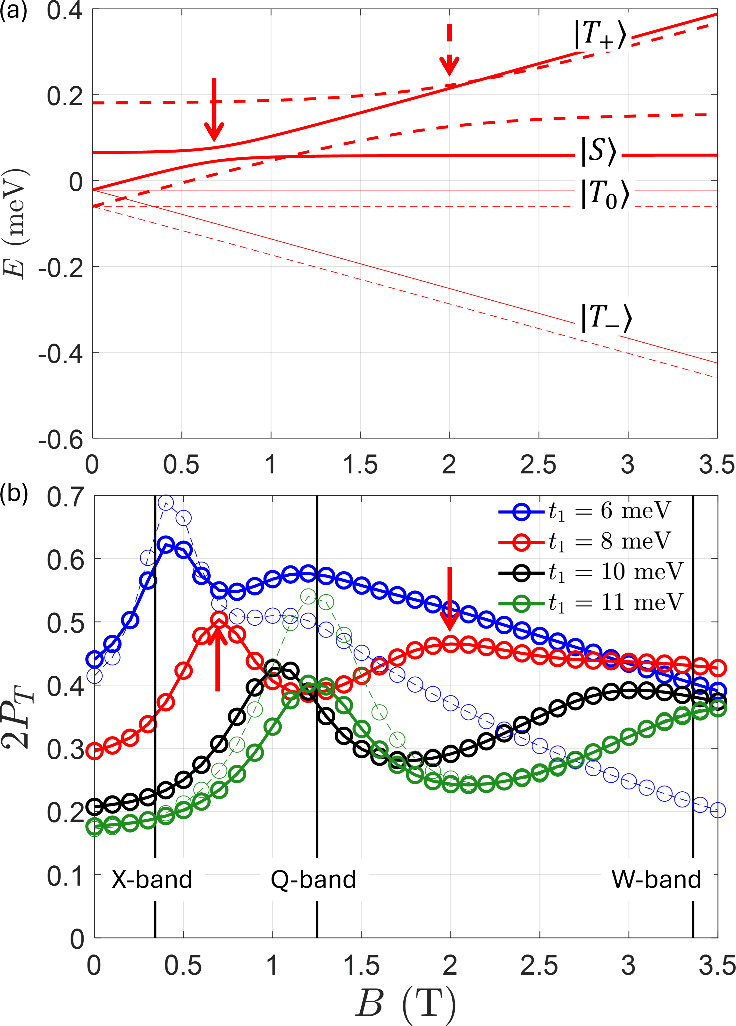
(a) Energy level diagram as a function of the magnetic field applied at θ = 90° with respect to the chiral anisotropy axis, using *t*_1_ = 8 meV, λ_1_ = 2.5 meV. Solid (dashed) curves refer to *n* = 0(1) boson, leading to AC at different fields. (b) CISS efficiency 2⟨*P*_*T*_⟩ at 80 K as defined in fitting EPR spectra. Different solid lines refer to different *t*_1_’s as indicated in the legend, while thin lines of the same color are obtained by doubling the boson energy while keeping the temperature fixed (ℏω = 2 → 4 meV). Dashed vertical lines indicate the fields probed by EPR at X-, Q-, and W-bands. The other parameters are kept fixed to *J*_*CE*_ = −1 × 10^–2^ meV, *t* = 1 meV, λ = 0.1 meV, *U* = 3.5 eV, Δ = 5 eV.

The corresponding CISS efficiency (represented by 2⟨*P*_*T*_⟩ in TREPR) is reported in Figure 3-(b) (red line and circles). Peaks are visible at each AC of panel (a), due to the increased mixing between |*S*⟩ and |*T*_+_⟩ when levels come close. ⟨*P*_*T*_⟩ also includes population of |*T*_–_⟩, which decreases due to the increasing Zeeman gap, while |*T*_0_⟩ is never populated in this axial model at θ = 90°. [In the liquid crystals alignment we are considering both |*T*_+_⟩ and |*T*_–_⟩ undergo an avoided level crossing for oppositely oriented sets of molecules.]

Other curves in Figure 3-(b) refer to different choice of *t*_1_, leading to ACs and corresponding peaks in 2⟨*P*_*T*_⟩ at different magnetic fields and of different widths. From Figure 3-(b) we immediately note that CISS efficiency comparable with experiments (in the 30%-60% range^6–8^ ) can be achieved in a realistic parameter range. Here the values of *t*_1_ are slightly higher than those employed in Figure 2, but perfectly realistic, since they account for the sum on several contributing modes (simulations for other parameter sets are reported in the Supporting Information). Moreover, depending on the parameters we can obtain different trends with magnetic fields. In particular, black and green curves show an efficiency doubled in going from X to Q-band (vertical lines) and only slightly reduced from Q to W band, as observed in DNA hairpins.^8^ Conversely, similar efficiency at the three probed bands is found for the red data set, in substantial agreement with ref 7. Besides changing *t*_1_ and λ_1_, a difference between CISS efficiencies at the various bands can be obtained also by varying the frequency of the vibrational mode, while keeping the temperature fixed. A few examples are represented by the thin blue and green lines in Figure 3-(b), where the frequency of the mode is doubled from 2 to 4 meV, thus making the two maxima sharper and yielding a more pronounced decrease with field for the blue curve. Considering higher-frequency modes further reduces the weight of thermally populated *n* > 0 sectors, yielding, for sufficiently large ℏω_ν_, a single sharper peak in the CISS efficiency as a function of *B*. Nonetheless, one can still obtain behaviors compatible with experiments^7^ or^8^ by different choices of the parameters, e.g. *t*_1_.

It is worth stressing that the value of ⟨*P*_*T*_⟩ (and hence the measured CISS efficiency and the related spin polarization) depends on the ratio between the Dzyaloshinskii-Moriya coupling *D*_*z*_ and the gap between singlet and triplet states. In zero field, this gap is determined by *J*, and hence a sizable ⟨*P*_*T*_⟩ requires λ_1_ comparable with *t*_1_. However, all EPR experiments are performed in applied field, where the singlet−triplet gap is tuned not only by *J* but also by the Zeeman energy. Consequently, experimental evidence of CISS is explained even for λ_1_ significantly smaller than *t*_1_, as demonstrated by simulations reported in Figure 3.

In general, we expect a nontrivial magnetic field dependence for most sets of parameters, apart from small *t*_1_ and *J*, leading to AC at very low field and thus an efficiency decreasing with *B*. For larger *t*_1_ (and hence larger *J* but still smaller than the Zeeman splitting in the Q/W band), at least one AC will occur at a higher magnetic field.

## Discussion and Future Experiments

We have introduced a vibrationally assisted mechanism explaining the observed triplet component in spin-correlated radical pairs generated by photoinduced electron transfer through a chiral bridge. Peierls vibrations give rise to an effective Dzyaloshinskii-Moriya interaction acting on the spin pair during the electron transfer and explain several seemingly conflicting observations: besides the large triplet component and spin polarization (i), the coexistence of large electronic energy gaps, giving rise to a single-electron picture of the electron-transfer process (ii) and of a low-energy dynamics yielding the measured magnetic-field dependence of the CISS efficiency (iii). Finally, the predicted polarization is robust with temperature (iv) as observed in several CISS experiments^3^ and shows a nontrivial temperature dependence that could be tested in future experiments. Here, the effect of temperature is to provide bosons to amplify the spin-Hamiltonian couplings. In principle, one could also incorporate an explicit temperature dependence of the electron-transfer rates, for example, within a Marcus-type formalism (as in ref 18). However, here we deliberately separate these effects to isolate the role of vibrationally induced spin interactions and to avoid introducing additional assumptions on the bath spectral density.

The interplay of energy scales between spin–spin coupling and Zeeman splitting leads to avoided level crossings in the spectrum that could be probed by tuning their position and width via the orientation of the molecules with respect to the magnetic field. Pulse EPR experiments could be employed to access the sensitivity to magnetic field fluctuations close to the avoided level crossings, which should yield maxima in the spin coherence times *T*_2_.^49^

As a benchmark of the present theory, one could design molecules in which the donor is a radical, thus reducing the transfer to a single-electron model and hence suppressing polarization. Alternatively, one could study molecules characterized by very large *static* (not vibrationally assisted) *J*, where any mixing of the singlet with the triplet should be suppressed. In this case we only expect a very small singlet–triplet imaginary coherence^17^ and a triplet component of the order of λ^2^/*t* ^2^. Molecules with chirality confined to the donor or to the acceptor could also provide an interesting benchmark of the theory: only the former should give a significant CISS effect. This is consistent with recent observation of reduced CISS efficiency in hole-transfer experiments in which chirality is confined to the donor (corresponding to the last ET step).^50^ Finally, to distinguish polarization and singlet–triplet imaginary coherence from the triplet population and discriminate the role of the two enantiomers we would need instead preparation of the sample with absolute orientation of the chiral molecules, as already discussed in ref 10.

For applications in Quantum Technologies (such as high-temperature initialization of a spin qubit or quantum sensing^10,51,52^ ), an important point to be discussed is the maximum achievable spin polarization. It can be easily demonstrated (see Supplementary Section V) that the maximum spin polarization accumulated on A is limited to 50% if we consider a monochromatic oscillating function on B transferred to A by an exponential decay rate. Note that this limit is dictated by the simple form of the sinusoidal oscillation on the bridge and hence it is not specific of the present model (see, e.g., ref 18). In fact, several possibilities to overcome the 50% polarization can be conceived, based on introducing additional harmonics in the coherent oscillations of the spin polarization on the bridge. The simplest option consists in increasing the length of the bridge by additional orbitals. [Coherent interorbital hopping can can be mapped onto the previous single-site description and requires very small hopping to overcome the 50% limit (see Supporting Information Section V).] A more realistic scenario is to consider a multistep incoherent hopping between multiple bridge sites coupled to D by exchange and DMI. In this case, large spin polarization can be achieved and can be systematically enhanced by adding more sites, as shown in Figure 4 and detailed in the Supporting Information.

**Figure 4:**
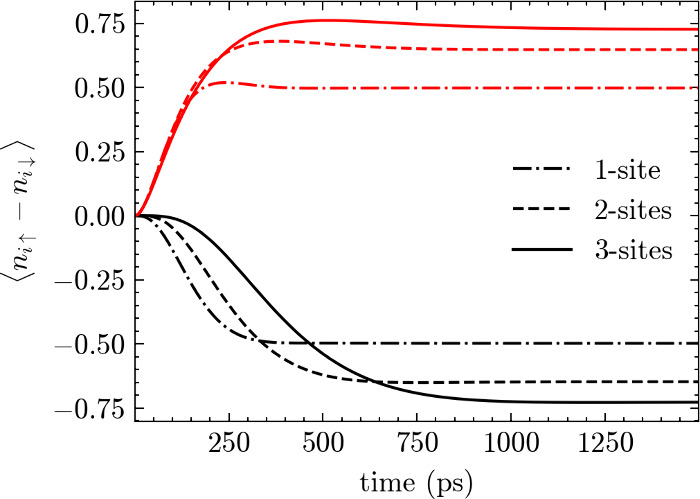
Evolution of the spin polarization on A (black) and D (red) for systems with one, two, and three sites on the bridge. Parameters are reported in Table 3 of the Supporting Information. Full population and spin polarization evolution are displayed in Figure S9.

The present results also provide a direct link between experiments performed in molecular electron transfer and on surfaces or junctions. Indeed, a DMI can arise also in chiral organic molecules hybridized with an underlying metal (such as Au), where surface magnetic moments were already observed,^36,53–55^ recalling the spinterface models.^37,56^

Moreover, vibrationally mediated DMI also arises from many-body descriptions of a chiral bridge.^21,22,28^ To demonstrate this point, we consider in the Supporting Information a tight-binding linear chain including hopping, SOC, and Peierls vibrations. Analogously to the results presented above, we find that Peierls modes modulating both hopping and SOC give rise to two-body interactions including isotropic, anisotropic, and antisymmetric exchange (proportional to *t*_1_^2^, λ_1_^2^, and λ_1_ *t*_1_).

Photoinduced donor–bridge–acceptor electron transfer realizes a controlled analogue of the interface scenario, with the crucial simplification of replacing the surface with a well-defined spin localized on the donor and with the molecular vibrations mediating spin–spin interactions, including a Dzyaloshinskii–Moriya term that arises due to SOC typical of chiral systems. Under this point of view, electron transfer is not disconnected from nanojunction experiments but rather represents a minimal setup in which the key spin degrees of freedom invoked in spinterface descriptions can be isolated and traced to microscopic molecular parameters.^57^ This makes donor–bridge–acceptor systems an ideal test bed for a microscopic theory of CISS relevant also to molecular spintronics, spin-selective photoemission, and surface-based transport.

In summary, the mechanism introduced here provides a physically transparent and robust route to spin polarization in photoinduced electron transfer through chiral systems. It explains the triplet component observed in EPR experiments and its magnetic field dependence, it offers several experimentally testable predictions, and it suggests synthetic strategies toward efficient CISS for applications in quantum technologies.

## Supplementary Material



## Notes

Details the derivation of eq 4, the Redfield framework, simulations on magnetic field sensitivity, strategies to bypass the 50% spin polarization limit, the methodology for the ab initio parametrization, and the derivation of vibrationally mediated interactions in a multielectron chiral bridge (PDF)
