# Supplementary material for: Vibrationally Mediated Dzyaloshinskii-Moriya Interaction as the Origin of Chirality-Induced Spin Selectivity in Donor–Acceptor Molecules
Source: Nano Lett. 2026 Jul 14;26(29):9465–72. doi: 10.1021/acs.nanolett.6c01653 (PMC13430671; doi:10.1021/acs.nanolett.6c01653)
Supplement: Supplementary file 1 [file nl6c01653_si_001.pdf]

Supporting Information to Vibrationally-mediated Dzyaloshinskii-Moriya interaction as the origin of Chirality-Induced Spin Selectivity in donor-acceptor molecules

Alessandro Chiesa,[1,2,3,*] D. K. Andrea Phan Huu,[1,†] Arianna Cantarella,[1,2,†] Leonardo Celada,[1,2] Michael R. Wasielewski,[4] Paolo Santini,[1,2,3] and Stefano Carretta[1,2,3,‡]

[1]*Dipartimento di Scienze Matematiche, Fisiche e Informatiche, Università di Parma, Parco Area delle Scienze, 53/A, I-43124 Parma, Italy.*

[2]*Gruppo Collegato di Parma, INFN-Sezione Milano-Bicocca, I-43124 Parma, Italy.*

[3]*UdR Parma, INSTM, I-43124 Parma, Italy.*

[4]*Department of Chemistry, Institute for Quantum Information Research and Engineering, and Center for Molecular Quantum Transduction, Northwestern University, Evanston, IL 60208-3113*

[*] alessandro.chiesa@unipr.it

[†] These authors contributed equally to this work.

[‡] stefano.carretta@unipr.it

I. DERIVATION OF THE SPIN-SPIN INTERACTIONS

Hereafter we derive the effective Hamiltonian H_{eff} of the main text. For sake of clarity, we start by considering only the electronic contribution and then we add the coupling to vibrations.

A. Electronic super-exchange

The *electronic* Hamiltonian describing a pair of electrons on the two orbitals D and j can be separated into a leading term H_0 and a perturbation H_1 given by

$$H = H_0 + H_1 \tag{S1a}$$

$$H_0 = U_D n_{D\uparrow} n_{D\downarrow} + U_j n_{j\uparrow} n_{j\downarrow} + \Delta(n_{j\uparrow} + n_{j\downarrow}) \tag{S1b}$$

$$\begin{aligned} H_1 &= -t \sum_\sigma (c^\dagger_{j\sigma} c_{D\sigma} + \text{h.c.}) + i\lambda(c^\dagger_{j\uparrow} c_{D\uparrow} - c^\dagger_{j\downarrow} c_{D\downarrow} - \text{h.c.}) \\ &= (-t + i\lambda) c^\dagger_{j\uparrow} c_{D\uparrow} + (-t - i\lambda) c^\dagger_{j\downarrow} c_{D\downarrow} + \text{h.c.} \\ &= \Lambda \left[e^{-i\varphi} c^\dagger_{j\uparrow} c_{D\uparrow} + e^{i\varphi} c^\dagger_{j\downarrow} c_{D\downarrow} + e^{i\varphi} c^\dagger_{D\uparrow} c_{j\uparrow} + e^{-i\varphi} c^\dagger_{D\downarrow} c_{j\downarrow} \right] \end{aligned} \tag{S1c}$$

where $\Lambda = \sqrt{t^2 + \lambda^2}$, $\varphi = \arccos t/\Lambda = \arcsin \lambda/\Lambda = \arctan \lambda/t$. We have included for simplicity only the z component of the spin orbit coupling, but this assumption does not qualitatively affect our conclusions. To be more general, we consider a generic site j on the bridge, coupled to the donor HOMO. Although in principle the Coulomb interaction can vary from site to site, we simplify the model by taking $U_j = U_D = U$.

To proceed, we can either eliminate H_1 to first order by a proper Schrieffer-Wolf transformation $H_{\text{eff}} = e^S H e^{-S}$ or apply second-order perturbation theory for $t, \lambda \ll U_D, \Delta$. The low energy manifold consists of the four localized spin states $c^\dagger_{D\sigma} c^\dagger_{j\sigma'} |\emptyset\rangle \equiv |\sigma\sigma'\rangle$, $\{\sigma, \sigma' = \uparrow, \downarrow\}$. The effective Hamiltonian in this low-energy subspace becomes

$$\langle\sigma\sigma'| H_{\text{eff}} |\sigma''\sigma'''\rangle = -\sum_k \frac{\langle\sigma\sigma'| H_1 |k\rangle \langle k| H_1 |\sigma''\sigma'''\rangle}{E_k}, \tag{S2}$$

where we have shifted to zero the energy of the low energy manifold and the excited states are $c^\dagger_{D\uparrow} c^\dagger_{D\downarrow} |\emptyset\rangle$ and $c^\dagger_{j\uparrow} c^\dagger_{j\downarrow} |\emptyset\rangle$, at energies $U - \Delta$ and $U + \Delta$. We find the following matrix

form for H_{eff}

$$\begin{pmatrix} 0 & 0 & 0 & 0 \\ 0 & -\frac{t^2+\lambda^2}{\Delta'} & \frac{t^2-\lambda^2-2i\lambda t}{\Delta'} & 0 \\ 0 & \frac{t^2-\lambda^2+2i\lambda t}{\Delta'} & -\frac{t^2+\lambda^2}{\Delta'} & 0 \\ 0 & 0 & 0 & 0 \end{pmatrix} \tag{S3}$$

with $1/\Delta' = 1/(U-\Delta) + 1/(U+\Delta)$. This effective spin Hamiltonian can be recast in the form

$$H_{\text{spin}} = J\mathbf{s}_1 \cdot \mathbf{s}_2 + J_D(2s_1^z s_2^z - s_1^x s_2^x - s_1^y s_2^y) + D_z(s_1^x s_2^y - s_1^y s_2^x) \tag{S4}$$

where the three contributions account for an isotropic, axial anisotropic and anti-symmetric exchange terms. Only the $q = 0$ components appear in Hamiltonian (S4) because we started from an axially symmetric Hamiltonian (S1). The values of the couplings are

$$J = \frac{2t^2 - 2\lambda^2/3}{\Delta'} \tag{S5a}$$

$$J_D = \frac{4\lambda^2}{3\Delta'} \tag{S5b}$$

$$D_z = \frac{4\lambda t}{\Delta'}. \tag{S5c}$$

B. Peierls vibrations

We now consider as a perturbation on H_0 coupling of the fermionic system with Peierls vibrations modulating both hopping and spin-orbit interactions, i.e. an Hamiltonian term of the form

$$H_{1P} = \sum_\nu (a_\nu + a_\nu^\dagger)[(t_{1\nu} + i\lambda_{1\nu})c_{\text{j}\uparrow}^\dagger c_{\text{D}\uparrow} + (t_{1\nu} - i\lambda_{1\nu})c_{\text{j}\downarrow}^\dagger c_{\text{D}\downarrow}] + \text{h.c.}, \tag{S6}$$

where we are considering several vibrational modes ν of energy $\hbar\omega_\nu$ coupled to the same fermionic terms. Note that a diagonal term in the number of bosons $\sum_\nu \hbar\omega_\nu a_\nu^\dagger a_\nu$ must be added to H_0.

We then consider the effect of H_{1P} by second-order perturbation theory analogously to the previous section ($t_1, \lambda_1 \ll \Delta - U$) and we derive an effective spin-spin interaction mediated by Peierls modes. The result an effective Hamiltonian of the same form of Eq. (S4), with

couplings:

$$J^P = 2\sum_\nu \left(t_{1\nu}^2 - \frac{\lambda_{1\nu}^2}{3}\right) f(n_\nu) \approx 2\sum_\nu \left(t_{1\nu}^2 - \frac{\lambda_{1\nu}^2}{3}\right) \frac{2n_\nu+1}{\Delta'} \tag{S7a}$$

$$J_D^P = \sum_\nu \frac{4\lambda_{1\nu}^2}{3} f(n_\nu) \approx \sum_\nu \frac{4\lambda_{1\nu}^2}{3\Delta'}\,(2n_\nu+1) \tag{S7b}$$

$$D_z^P = -4\sum_\nu \lambda_{1\nu} t_{1\nu} f(n_\nu) \approx -\sum_\nu \frac{4\lambda_{1\nu} t_{1\nu}}{\Delta'}\,(2n_\nu+1). \tag{S7c}$$

with

$$f(n_\nu) = \frac{n_\nu+1}{U_D-\Delta+\hbar\omega_\nu} + \frac{n_\nu+1}{U_j+\Delta+\hbar\omega_\nu} + \frac{n}{U_D-\Delta-\hbar\omega_\nu} + \frac{n_\nu}{U_j+\Delta-\hbar\omega_\nu} \approx \frac{2n_\nu+1}{\Delta'} \tag{S8}$$

and n_ν is the number of bosons of energy $\hbar\omega_\nu$. Note that since $|\Delta'| \gg \hbar\omega_\nu$ the result does not depend significantly on $\hbar\omega_\nu$.

Remarkably, the perturbative expansion converges even for $t_{1\nu}, \lambda_{1\nu} > \hbar\omega_\nu$, as long as

$$\frac{t_{1\nu}}{\Delta'}\frac{t_{1\nu}}{2\hbar\omega_\nu} n_\nu \ll 1. \tag{S9}$$

Since $t_{1\nu}/\Delta' \sim 10^{-2} - 10^{-3}$, we we can safely investigate regimes with $t_{1\nu}, \lambda_{1\nu} \sim 10\hbar\omega$ using the effective Hamiltonian derived above. Indeed, all odd orders are zero and the subsequent (fourth order) correction is $\sim t_{1\nu}^2 n_\nu/\Delta'\hbar\omega_\nu$ smaller than the second order one. This conclusion is supported by numerical simulations in Fig. 1 of the main text, showing very good agreement between simulations performed with H and $H_{\rm eff}$.

Note that in principle also mixed terms in H_1 and H_{1P} could give a second-order correction to the spectrum of H_0. However, these terms mix states with different number of bosons with off-diagonal terms of the order of J^P, J_D^P, D_z^P. Since these terms are $\ll \hbar\omega_\nu$, they do not impact significantly the dynamics (their effect being of the order of the difference between the dashed and solid lines in Fig. 1 c,d).

C. Summary of contributions to $H_{\rm eff}$

In summary, we have three contributions to the exchange: direct Coulomb (J_{CE}), electronic super-exchange and vibrationally mediated. The three contribution to J are typically all ferromagnetic (FM) due to the negative sign of Δ' (the vibrationally mediated term is FM for $t_1 \geq \lambda_1/\sqrt{3}$).

As discussed in the main text, the vibrationally-mediated contributions are the leading ones, because of the large values that $t_{1\nu}$ and $\lambda_{1\nu}$ can assume in chiral molecules, of the sum on different modes and of the $(2n_\nu + 1)$ factor which becomes relevant especially for modes at low energies (a few meV) compared to $k_B T$.

For computational reasons we can include only a single vibrational mode in our simulations, with the caveat that the assumed value of λ_1 and t_1 are *effective couplings* which take into account contributions from several modes.

Therefore, we obtain the following overall expressions for the spin Hamiltonian parameters

$$J \approx \frac{2}{\Delta'} \left[t^2 + (t_1^2 - \lambda_1^2/3)(2n+1)\right] + J_{CE} \tag{S10a}$$

$$D_z \approx \frac{4}{\Delta'} \left[\lambda t + \lambda_1 t_1 (2n+1)\right] \tag{S10b}$$

$$J_D \approx \frac{4\lambda_1^2}{3\Delta'}(2n+1), \tag{S10c}$$

where we have dropped the index ν and considered only a single coupled mode.

II. REDFIELD MASTER EQUATION

To describe the electron-transfer (ET) dynamics, we consider an interaction between the system and the bath of the form

$$H_{SB} = \sum_r \sum_{\nu=D,A} \kappa_{r,\nu}(X_\nu + X_\nu^\dagger)\,(a_{r,\nu} + a_{r,\nu}^\dagger) \tag{S11}$$

with the operators $X_D = \sum_\sigma c_{1\sigma}^\dagger c_{D\sigma}$ and $X_A = \sum_\sigma c_{A\sigma}^\dagger c_{i=4,\sigma}$ inducing electron hopping from the donor excited orbital onto the bridge or from the bridge to the acceptor, respectively. In Section II A below, we also account for a SOC contribution to the incoherent transfer from donor to bridge (see (S13) for the respective system-bath coupling operators).

Here $a_{r,\nu}$ is the bosonic annihilation operator for the r-th mode of the bath coupled with a strength $\kappa_{r,\nu}$ to either X_D or X_A. X_ν are rank-0 fermionic operators which do not affect the spin of the transferred electron. For simplicity, we do not include further coupling terms between the system and the bath.

We consider temperatures much smaller than the energy gaps driving ET and we describe

the time evolution of the system density matrix ρ by the Redfield equation [1]:

$$\hbar \frac{d\rho}{d\tau} = -i[H,\rho] + \sum_{\xi=D,A} \Gamma_\xi \left(Y_\xi \rho X_\xi^\dagger - X_\xi^\dagger Y_\xi \rho + \text{h.c.} \right). \tag{S12}$$

The first term of Eq. (S12) describes the coherent evolution induced by the Hamiltonian H, while $Y_\xi = \sum_{\mu,\nu} |\psi_\mu\rangle \langle\psi_\nu| \langle\psi_\mu| X_\xi |\psi_\nu\rangle D_{\mu,\nu}$ and $D_{\mu,\nu}$ are proportional to the bath spectral function and to the Bose-Einstein factor at the energy gap $E_\nu - E_\mu$. In the low-temperature and wide-band limits considered hereafter $D_{\mu,\nu} = \Theta(E_\nu - E_\mu)$. Finally, Γ_ξ are the system-bath coupling strengths (or friction coefficients). Unless otherwise noted, we set $\Gamma_D = \Gamma_A = \Gamma$. The master equation used here amounts to an effective Markovian hopping equation for the electronic density matrix. While typically the Redfield master equation is considered to give a poor description of the electron transfer due to the strong coupling between electronic and bath degrees of freedom, the same effective master equation was derived starting by the assumption of strong coupling, as e.g. in Ref.2 and 3.

A. Spin-orbit assisted incoherent transfer

Here we address the effect of SOC in the incoherent transfer from donor to bridge. Numerical simulations (Fig. S1 and Fig. S2) are performed with the set of parameters reported in Table I.

Spin-orbit assisted incoherent transfer can be introduced in two ways

1. Hopping and SOC are both coupled to the same bath. In this case, in order to keep the overall transfer rate equal, the system-bath coupling operator is given by

$$\hat{X}_D = \cos\theta \sum_\sigma c_{1\sigma}^\dagger c_{D\sigma} + i \sin\theta \sum_{\sigma,\sigma'} c_{1\sigma}^\dagger \sigma_{\sigma\sigma'}^z c_{D\sigma'} \tag{S13}$$

2. Hopping and SOC are coupled to different baths. In this case, the system bath coupling Hamiltonian (S11) becomes

$$H_{SB} = \sum_r \sum_{\nu=D,\tilde{D},A} \kappa_{r,\nu} (X_\nu + X_\nu^\dagger)\, (a_{r,\nu} + a_{r,\nu}^\dagger) \tag{S14}$$

with

$$\hat{X}_{\tilde{D}} = i \sum_{\sigma,\sigma'} c_{1\sigma}^\dagger \sigma_{\sigma\sigma'}^z c_{D\sigma'} \tag{S15}$$

The master equation S12 becomes

$$\hbar\frac{d\rho}{d\tau} = -i[H,\rho] + \sum_{\xi=D,\tilde{D},A} \Gamma_\xi \left(Y_\xi \rho X_\xi^\dagger - X_\xi^\dagger Y_\xi \rho + \text{h.c.} \right). \tag{S16}$$

to keep the overall transfer rate equal we define the total transfer rate from donor to acceptor as

$$\Gamma_{D,tot} = \Gamma_D + \Gamma_{\tilde{D}} \tag{S17}$$

The inclusion of a small incoherent SOC contribution for the donor-to-bridge transfer following approach (1) has negligible effects on ET dynamics and spin polarization (see left and central panels of Fig. S1). For large (and arguably unrealistic) SOC, the spin polarization is reduced as displayed in the right panel of Fig. S1, where a 2:1 ratio between hopping and SOC is imposed.

Following approach (2) we obtain qualitatively similar results. When the incoherent transfer from donor to bridge is partially due to SOC, the spin polarization accumulated on the acceptor is reduced. This effect is small, albeit non-negligible, when $\Gamma_{\tilde{D}}/\Gamma_D = 1/10$) and more pronounced when the SOC contribution is large, as displayed in Fig. S2.

Table I: Microscopic parameters and the resulting effective spin-coupling parameters for Fig. S1 and Fig. S2.

**Microscopic parameters (meV)**	
t	1.0
t_1	1.8
λ	0.1
λ_1	2.1
U	3500
Δ	5000
$\hbar\omega$	2.0
J_{CE}	0.001
n_{ph}	0

**Effective spin-spin parameters (meV)**	
J	$-4.04 \cdot 10^{-3}$
J_D	$-3.24 \cdot 10^{-3}$
D_z	$-8.53 \cdot 10^{-3}$

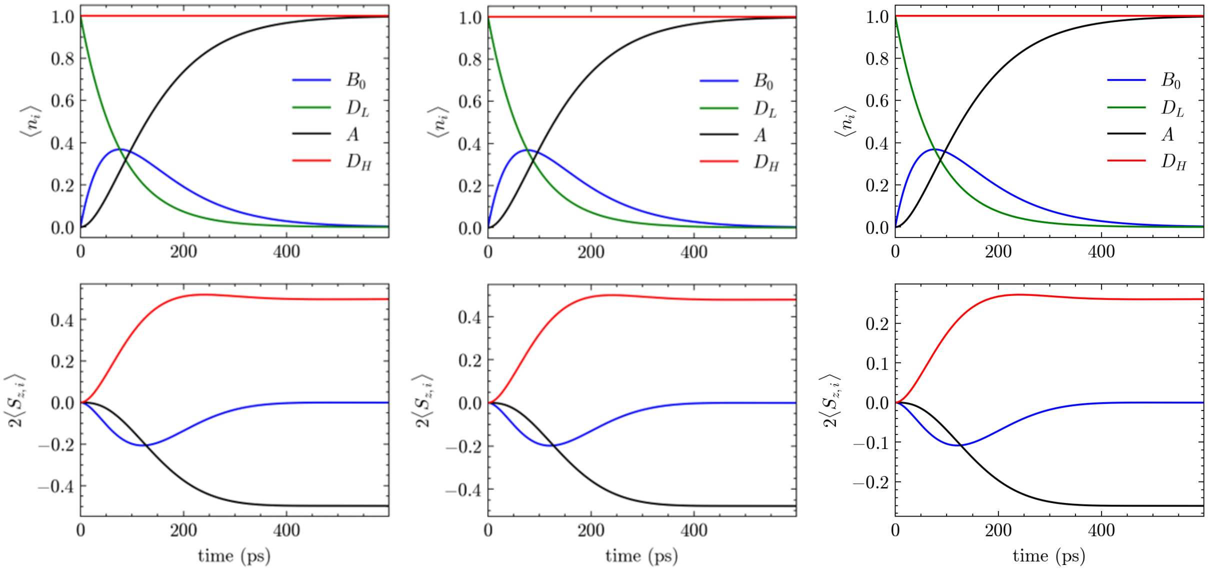

Figure S1: Population and spin polarization evolution accounting for spin-orbit assisted incoherent transfer from donor to bridge, according to approach 1. Parameters are set as describe in Section II A. The transfer rates are set as $\Gamma_{D,tot} = \Gamma_A = 4.3 \times 10^{-3}$ meV, and different columns refer to different values of θ. Left: $\theta = 0$. Center: $\theta = \arctan(0.1)$. Right: $\theta = \arctan(0.5)$.

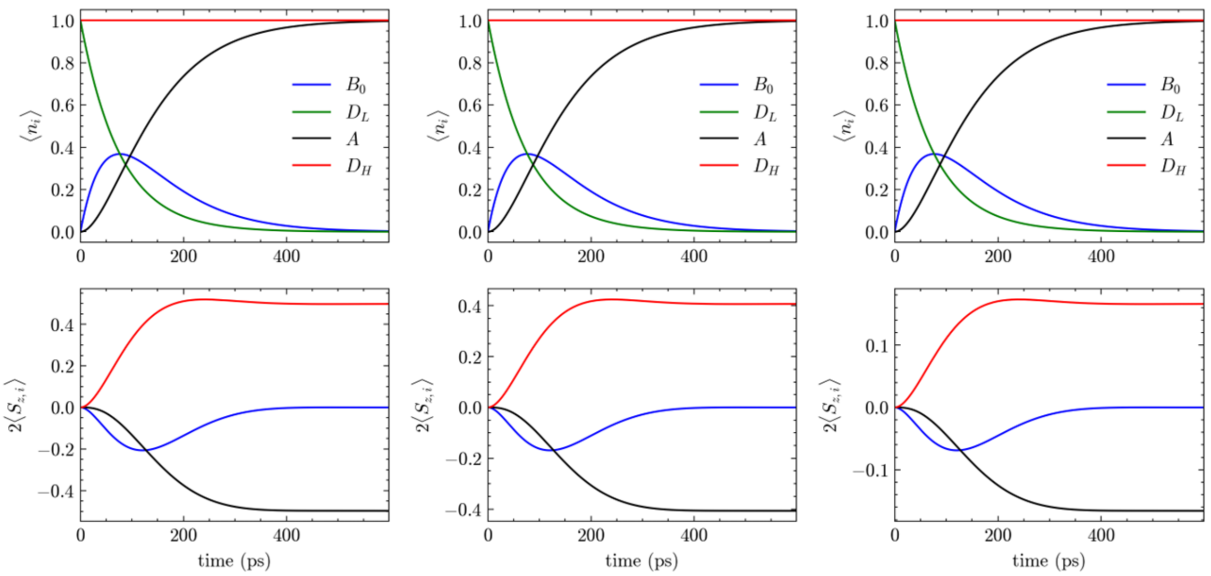

Figure S2: Population and spin polarization evolution accounting for spin-orbit assisted incoherent transfer from donor to bridge, according to approach 2. Parameters are set as described in Section II A. The transfer rates are set as $\Gamma_{D,tot} = \Gamma_A = 4.3 \times 10^{-3}$ meV, and different columns refer to different $\Gamma_{\tilde{D}}/\Gamma_D$ ratios. Left: $\Gamma_{D,tot} = \Gamma_D$. Center: $\Gamma_{\tilde{D}}/\Gamma_D = 1/10$. Right: $\Gamma_{\tilde{D}}/\Gamma_D = 1/2$.

B. Vibrational damping

Vibrational damping is introduced in the master equation framework via jump operators a_v and $a_v^\dagger$ to describe vibrational loss and vibrational gain, respectively, so that the master equation (S12) becomes

$$\hbar \frac{d\rho}{d\tau} = -i[H,\rho] + \sum_{\xi=D,A} \Gamma_\xi \left(Y_\xi \rho X_\xi^\dagger - X_\xi^\dagger Y_\xi \rho + \text{h.c.} \right) + \tag{S18}$$

$$+ \sum_{\alpha=c,d} \gamma \left(A_\alpha \rho B_\alpha^\dagger - B_\alpha^\dagger A_\alpha \rho + \text{h.c.} \right) \tag{S19}$$

where $H = H_0 + H_1$ from (3) in the main text, $B_c = a^\dagger$ and $B_d = a$, $A_\alpha = \sum_{\mu\nu} |\psi_\mu\rangle \langle\psi_\nu| \langle\psi_\mu| B_\alpha |\psi_\nu\rangle D_{\mu\nu}$, $D_{\mu\nu}$ is proportional to the bath spectral function and Bose-Einstein factor at the energy gap $E_\nu - E_\mu$, and γ is the damping rate.

To avoid the need of renormalizing all the model parameters when the damping changes the effective spin-spin couplings, we focus on a low-temperature case, by considering a mode whose energy is significantly larger than $k_B T$. In this limit we can study the effect of phonon loss with the same effective parameters of Fig. (1). The local spin polarization on the acceptor for different values of the damping rate γ is reported in Fig. S3. The spin polarization is only weakly affected by vibrational damping, even in the overdamped regime ($\gamma = 10 - 100$ meV).

To understand the effect of vibrational damping, it is useful to consider a simplified model with three electronic states $|S\rangle = \frac{1}{\sqrt{2}} \left(c_{D\uparrow}^\dagger c_{1\downarrow}^\dagger - c_{D\downarrow}^\dagger c_{1\uparrow}^\dagger \right) |\emptyset\rangle$, $|T\rangle = \frac{1}{\sqrt{2}} \left(c_{D\uparrow}^\dagger c_{1\downarrow}^\dagger + c_{D\downarrow}^\dagger c_{1\uparrow}^\dagger \right) |\emptyset\rangle$, and $|D\rangle = c_{D\uparrow}^\dagger c_{D\downarrow}^\dagger |\emptyset\rangle$ and a small vibrational space of $n = 0, 1$. In the perturbative regime ($t_1, \lambda_1 \ll \Delta'$), the first order corrected singlet and triplet wavefunctions are

$$|\tilde{S0}\rangle \approx |S0\rangle + \frac{t_1}{\Delta'} |D1\rangle \tag{S20}$$

$$|\tilde{T0}\rangle \approx |T0\rangle + i\frac{\lambda_1}{\Delta'} |D1\rangle \tag{S21}$$

$$|\tilde{S1}\rangle \approx |S1\rangle + \frac{t_1}{\Delta'} |D0\rangle + \sqrt{2}\frac{t_1}{\Delta'} |D2\rangle \tag{S22}$$

$$|\tilde{T1}\rangle \approx |T1\rangle + i\frac{\lambda_1}{\Delta'} |D0\rangle + i\sqrt{2}\frac{\lambda_1}{\Delta'} |D2\rangle \tag{S23}$$

The phonon loss operator matrix elements in the $\{\tilde{Sn}, \tilde{Tn}\}$ subspaces are zero at any order.[4]

The largest matrix elements of a coupling states in the 0 and 1 subspaces are spin-preserving,

$$\langle\tilde{S0}|\, a\, |\tilde{S1}\rangle \approx 1 + \sqrt{2}\frac{t_1^2}{\Delta'^2} \tag{S24}$$

$$\langle\tilde{T0}|\, a\, |\tilde{T1}\rangle \approx 1 - \sqrt{2}\frac{\lambda_1^2}{\Delta'^2} \tag{S25}$$

and introduce relaxation from vibrationally excited subspaces to the vibrationally thermal state. This process is relatively fast for low frequency modes in the overdamped regime, ($\gamma > \hbar\omega$). However, a vibrational thermal state is unaffected by vibrational damping, so that the temperature dependence of spin polarization shown in Fig. 2, computed at the vibrational thermal equilibrium is not affected by this direct vibrational relaxation.

A more subtle effect is due to the matrix elements of a of the type

$$\langle\tilde{D0}|\, a\, |\tilde{S0}\rangle \approx \frac{t_1}{\Delta'} \tag{S26}$$

$$\langle\tilde{D0}|\, a\, |\tilde{T0}\rangle \approx i\frac{\lambda_1}{\Delta'} \tag{S27}$$

Indeed, since we are in a strongly perturbative regime ($t_1, \lambda_1 \ll \Delta'$), vibrational modes are only virtually excited to mediate an effective spin-spin interaction. In other words, only a small fraction of the low-energy wavefunction of the order of t_1/Δ' is subject to damping. As a result, the effective damping rate acting on the spin subspace is reduced by a factor $t_1/\Delta' \sim 10^{-3}$. Hence, we expect a qualitative effect of damping on spin polarization only for $\gamma t_1/\Delta'$ of the order of the Peierls coupling, i.e. $\gamma \sim \Delta' \sim$eV. The same results are obtained in a more formal approach by applying the Lang-Firsov transformation to the phonon loss operator, as displayed in Section VIII.

III. DEPENDENCE ON THE MODULATED PARAMETERS AND ET RATE

We show below a multi-panel colormap analogous to Fig. 2 of the main text, but assuming a faster ET dynamics.

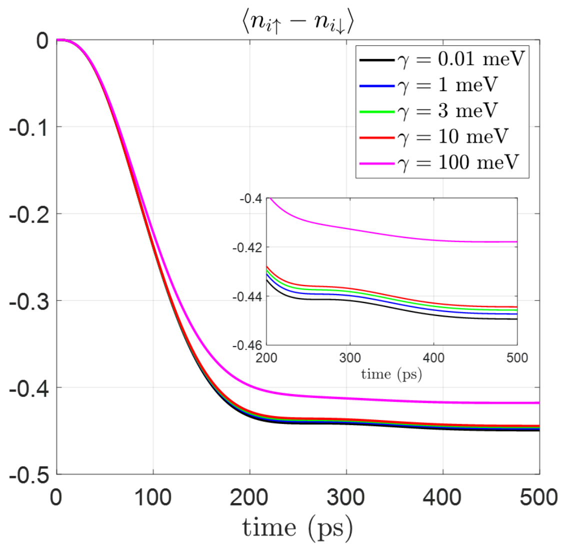

Figure S3: Simulated time evolution of the local spin polarization on the acceptor subunit accounting for different vibrational damping rates at zero temperature, according to (S18), where $H = H_0 + H_1$ (see (3)). The same model parameters as in the caption of Fig. 1 are used.

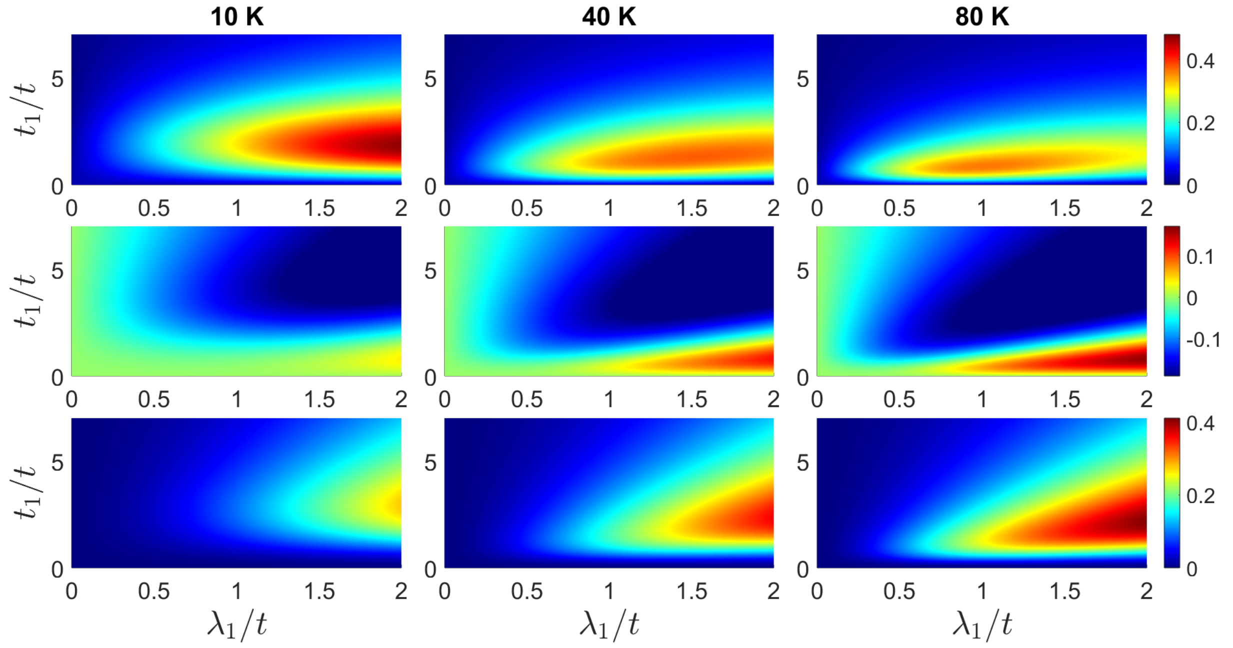

Figure S4: Top line: spin polarization; middle: imaginary singlet-triplet coherence; bottom: triplet component. Parameters as in Fig. 2 of the main text, extended range of t_1.

IV. FIELD-DEPENDENCE

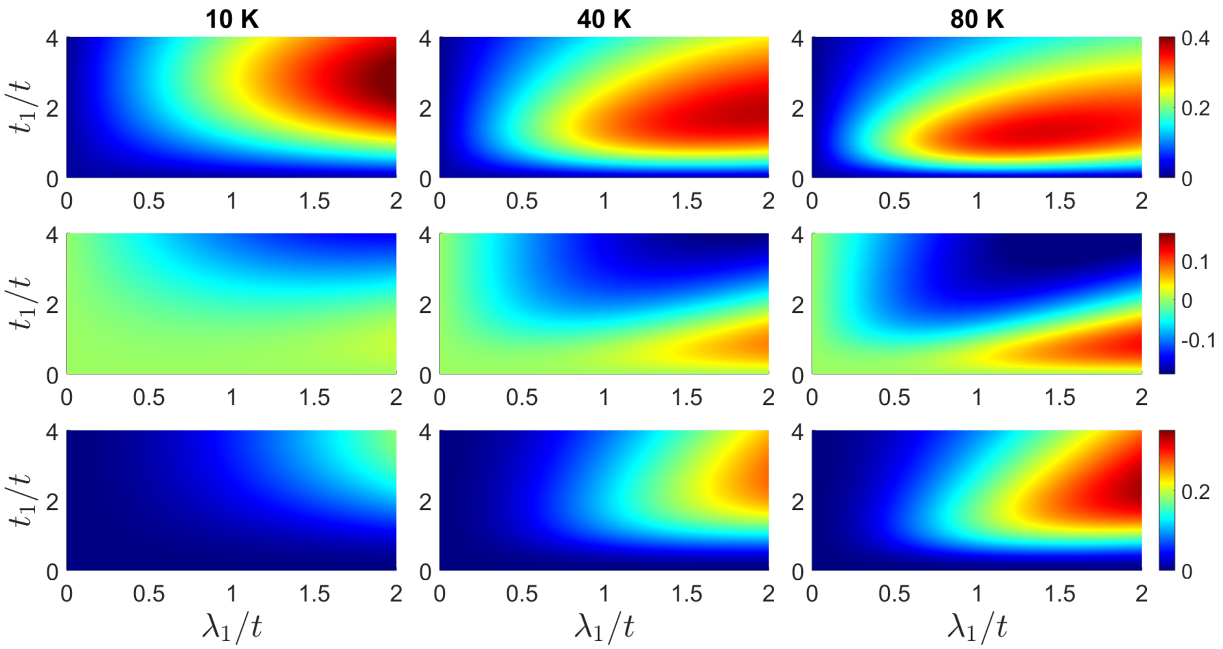

Figure S5: Top line: spin polarization; middle: imaginary singlet-triplet coherence; bottom: triplet component. Parameters as in Fig. 2 of the main text, but with faster ET ($\Gamma = 1\times10^{-2}$ meV).

V. MAXIMUM ACHIEVABLE SPIN POLARIZATION

A. Origin of the 50% limit in simplest models

We consider a minimal kinetic model involving four states: $c^\dagger_{B\uparrow}|\emptyset\rangle$, $c^\dagger_{B\downarrow}|\emptyset\rangle$, $c^\dagger_{A\uparrow}|\emptyset\rangle$, $c^\dagger_{A\downarrow}|\emptyset\rangle$, where B, A represent bridge and acceptor orbitals. The four states correspond to an electron on B and A with spin $\uparrow$ or $\downarrow$. In the following we adopt the notation $B_\sigma = \langle c^\dagger_{B\sigma} c_{B\sigma}\rangle$, with $\sigma =\uparrow,\downarrow$. In the absence of coupling to the acceptor, we consider a spin polarization on the bridge site evolving periodically as:

$$s_{zB}(t) = B_\uparrow(t) - B_\downarrow(t) = a\sin(\omega t) \tag{S28}$$

where $0 \leq a \leq 1$ represents the amplitude of the oscillating polarization and ω its frequency. The choice of a sinusoidal form for the spin polarization on the bridge site is dictated by the fact that (i) it satisfies the physical constraint that the initial spin polarization is null, i.e., $B_\uparrow(0) = B_\downarrow(0) = 1/2$, (ii) in a coherent regime where two states are coupled, the polarization must evolve as a monochromatic oscillation, with ω proportional to the energy gap of the

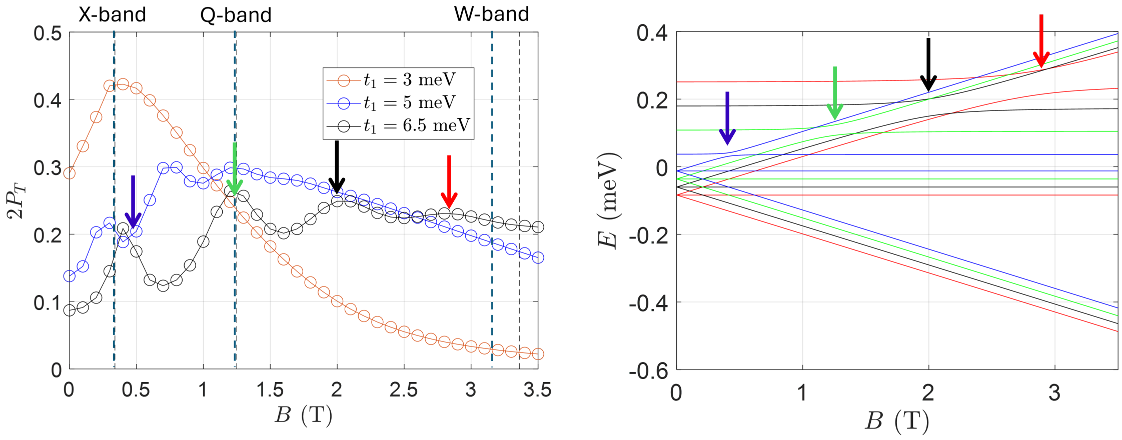

Figure S6: Left: field-dependence of the triplet component at 80 K, $\theta = 90°$ between chiral anisotropy axis and external field, with parameters as in Fig. 2 of the main text, $\lambda_1 = 1$ meV and different t_1 as indicated in the legend. Right: level diagram for $t_1 = 6.5$ meV, with different colors referred to different $n = 0, 1, 2, 3$ and avoided level crossing marked by arrows corresponding to each peak in the triplet component. At larger n the coupling increases, yielding a corresponding broadening of the avoided crossing and of the peak in P_T.

spin eigenstates, and (iii) when $0 \leq a \leq 1$ the sine function ensures that the polarization never exceeds the total population.

We now include an irreversible population transfer from B to A at a rate Γ. The time evolution of the populations is governed by the following system of kinetic equations:

$$\begin{cases} \dot{A}_\uparrow = \Gamma B_\uparrow \\ \dot{A}_\downarrow = \Gamma B_\downarrow \\ \dot{B}_\uparrow = -\Gamma B_\uparrow \\ \dot{B}_\downarrow = -\Gamma B_\downarrow \end{cases} \tag{S29}$$

Assuming that at $t = 0$ the entire population is on the bridge and since the transfer is spin independent, the population on the bridge decays as $B_\sigma(t) = B_\sigma(0)e^{-\Gamma t}$. Consequently, the time-dependent spin polarization on the bridge site is given by:

$$s_{zB}(t) = B_\uparrow(t) - B_\downarrow(t) = ae^{-\Gamma t}\sin(\omega t) \tag{S30}$$

The rate of change for the spin polarization on the acceptor site A is then:

$$\dot{s}_{zA}(t) = \dot{A}_\uparrow(t) - \dot{A}_\downarrow(t) = \Gamma\left[B_\uparrow(t) - B_\downarrow(t)\right] = \Gamma ae^{-\Gamma t}\sin(\omega t). \tag{S31}$$

By integrating this expression over time, we obtain the time dependence of the spin polarization at site A:

$$s_{zA}(t) = A_{\uparrow}(t) - A_{\downarrow}(t) = \Gamma a \int_0^t e^{-\Gamma\tau} \sin(\omega\tau)\, d\tau \tag{S32}$$

Solving the integral yields the analytical expression for the acceptor spin polarization:

$$s_{zA}(t) = \frac{\Gamma a}{\Gamma^2 + \omega^2} \left[\omega - e^{-\Gamma t}\big(\omega \cos(\omega t) + \Gamma \sin(\omega t)\big)\right] \tag{S33}$$

In the long-time limit ($t \to \infty$), the steady-state spin polarization on the acceptor reaches:

$$s_{zA}(\infty) = \frac{\Gamma a \omega}{\Gamma^2 + \omega^2} \tag{S34}$$

To determine the maximum achievable polarization, we analyze the function $f(\Gamma, \omega) = \frac{\Gamma\omega}{\Gamma^2+\omega^2}$. By setting the derivative with respect to ω to zero:

$$\frac{df}{d\omega} = \frac{\Gamma(\Gamma^2 + \omega^2) - 2\Gamma\omega^2}{(\Gamma^2 + \omega^2)^2} = \frac{\Gamma(\Gamma^2 - \omega^2)}{(\Gamma^2 + \omega^2)^2} = 0 \tag{S35}$$

we find that the maximum occurs when $\Gamma = \omega$ (assuming $\Gamma > 0$ for a non-trivial transfer). Under this condition, the maximum polarization is:

$$s_{zA}(\infty, \Gamma = \omega) = \frac{a \cdot \Gamma^2}{2\Gamma^2} = \frac{a}{2} \leq \frac{1}{2} \tag{S36}$$

where $s_{zA}(\infty, \Gamma = \omega)$ is limited by the amplitude parameter $a \leq 1$.

B. Beyond the 50% limit: formal analysis

To overcome this 50% limit, the system should either deviate from the initial assumption of considering a sinusoidal form for the spin polarization on the bridge site or dropping the assumption of monoexponential population transfer. This latter scenario can be physically obtained with non-Markovian baths.

Hereafter we prefer to keep the simplest assumption of a Markovian bath leading to an exponential incoherent transfer and focus on the generation of poly-chromatic oscillations of s_{zB}. From an analytical perspective, overcoming the 50% limit requires a spin polarization form that rises more steeply than the sine wave and remains near its maximum value for longer times. On the other side, the initial constraints (i) and (iii) detailed in Supplementary

Section V A must be preserved. A way to satisfy these conditions is to assume a square-wave like profile, which can be represented analytically through a Fourier series of odd harmonics:

$$s_{zB}(t) = e^{-\Gamma t} \frac{4a}{\pi} \sum_{n=1,3,5,\ldots}^{\infty} \frac{1}{n} \sin(n\omega t) \tag{S37}$$

Following the same reasoning of the previous section, the polarization at site A becomes:

$$s_{zA}(t) = \frac{4\Gamma a}{\pi} \sum_{n=1,3,5,\ldots}^{\infty} \frac{1}{n} \int_0^t e^{-\Gamma\tau} \sin(n\omega\tau)\, d\tau \tag{S38}$$

Since we are interested in the polarization accumulated over long times, and the integrand decays exponentially, we can extend the upper limit of integration to $+\infty$. The integral then separates into n independent terms, each of the standard Laplace form: $I_n = \int_0^\infty e^{-\Gamma\tau} \sin(n\omega\tau)$ which has as solution $I_n = \frac{n\omega}{\Gamma^2+(n\omega)^2}$. The resulting polarization becomes:

$$s_{zA}(t) = \frac{4\Gamma a}{\pi} \sum_{n=1,3,5,\ldots}^{\infty} \frac{1}{n} \frac{n\omega}{\Gamma^2 + (n\omega)^2} = \frac{4\Gamma a\omega}{\pi} \sum_{n=1,3,5,\ldots}^{\infty} \frac{1}{\Gamma^2 + (n\omega)^2} \tag{S39}$$

To study the convergence of the series, we can use the standard result $\sum_{n=0}^{\infty} \frac{1}{(2n+1)^2+x^2} = \frac{\pi}{4x} \tanh\left(\frac{\pi x}{2}\right)$ with $x = \frac{\Gamma}{\omega}$:

$$s_{zA}(\infty) = \frac{4\Gamma a}{\pi\omega} \sum_{n=0}^{\infty} \frac{1}{(2n+1)^2 + (\Gamma/\omega)^2} = \frac{4\Gamma a}{\pi\omega} \frac{\pi\omega}{4\Gamma} \tanh\left(\frac{\pi\Gamma}{2\omega}\right) = a \tanh\left(\frac{\pi\Gamma}{2\omega}\right) \tag{S40}$$

In this case, for $\omega = \Gamma$ the value of $s_{zA}(\infty)$ is over $0.9a$, by far exceeding the 50% limit. A more physical scenario consists of considering only the first harmonics of the series in Eq. S37, corresponding to a system in which the donor interacts with a few bridge sites oscillating, e.g., at frequencies $\omega, 3\omega, 5\omega$. For the truncated series, we drop the $\frac{4}{\pi}$ normalization factor. Keeping the normalization factor would cause the truncated profile to overshoot a, producing unphysical values of the spin polarization on the bridge sites. As a consequence, the spin polarization we find at longer times for this three-frequencies example represents a lower bound on what the properly normalized truncated series would yield. Yet, although this truncation is far from the ideal square-wave limit, it already gives a substantial improvement over the single-frequency case. We obtain the following spin polarization at longer times on the acceptor:

$$s_{zA}(\infty) = a \left[\frac{\Gamma\omega}{\Gamma^2 + \omega^2} + \frac{\Gamma\omega}{\Gamma^2 + 9\omega^2} + \frac{\Gamma\omega}{\Gamma^2 + 25\omega^2} \right] \tag{S41}$$

which is maximized by taking $\Gamma/\omega = 1.31$, resulting in $s_{zA}(\infty)_{\Gamma=1.31\omega} = 0.65\, a$. This derivation demonstrates that any physical mechanism yielding a polychromatic oscillation on the

last site of the bridge (with a steep increase followed by a flat region) can be exploited to overcome the 50% limit.

C. Physical mechanisms to overcome the 50% limit: coherent hopping

Perhaps the easiest way to generate multi-frequency coherent oscillations in the spin polarization of the bridge sites is to extend the model described in the main text to account for more than one site on the bridge. As a proof of concept, we compare the spin dynamics for a one-bridge-site model (Fig. S7a) with those obtained for models consisting of two bridge-sites (Fig. S7b and Fig. S7c). The microscopic parameters (Table I and Table II) are chosen so that, with the appropriate transfer rate $\Gamma = 4.3 \times 10^{-3}$ meV, $\approx 50\%$ spin polarization is accumulated on A for a system with one site on the bridge.

In the case of two or more bridge sites, the one-electron Hamiltonian for the bridge is

$$H_{bridge} = \sum_{i=0}^{N} \sum_{\sigma} \varepsilon_i c^{\dagger}_{i,\sigma} c_{i,\sigma} + \sum_{i,j \neq i} \sum_{\sigma} t_{ij} c^{\dagger}_{i,\sigma} c_{j,\sigma} + h.c. \tag{S42}$$

Where N is the number of sites on the bridge, ε_i is the on-site energy, and t_{ij} is the hopping integral between sites i and j. Specifically, in the simulations from Fig. S7b and c, $t_{01} = 0.1$ meV and $\varepsilon_0 = \varepsilon_1$.

The addition of a second bridge site, uncoupled to the donor spin, allows one to overcome the 50% limit on spin polarization on the acceptor. This is evident in Fig. S8 and from the comparison between Fig. S7a and Fig. S7b, where the static spin polarization on the acceptor goes from $\approx 50\%$ to $\approx 55\%$. A similar enhancement is observed when a finite spin–spin coupling between B_1 and the donor is included (Fig. S7c).

D. Physical mechanisms to overcome the 50% limit: incoherent transfer

Spin polarization over 50% can also be achieved exploiting multistep incoherent transfer among bridge sites spin-coupled to the donor. We consider a travelling electron moving through a chain of n bridge sites (B_i) towards an acceptor site (A):

$$B_1 \xrightarrow{\Gamma_1} B_2 \xrightarrow{\Gamma_2} \cdots \xrightarrow{\Gamma_n} A$$

Specifically, we assume here incoherent transfer, with Γ_i being the rate for transfer from site B_i to site B_{i+1}. While on site B_i, the electron is spin-coupled to a second electron

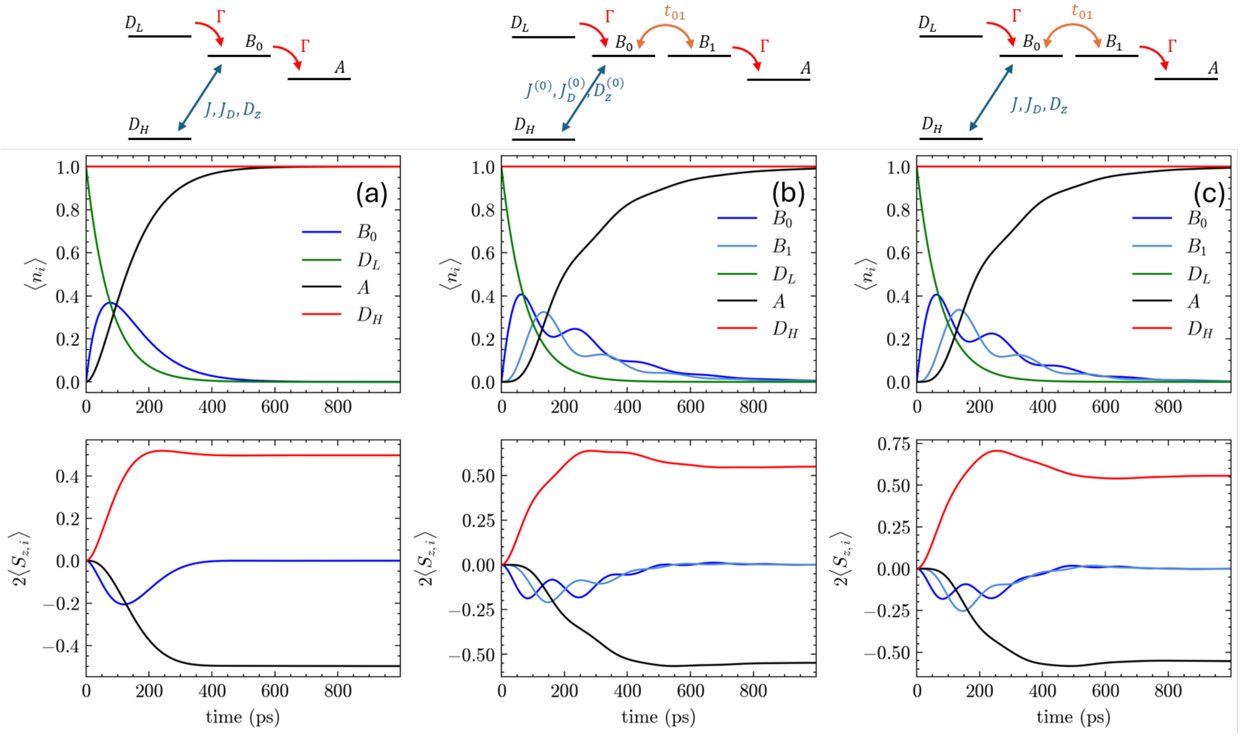

Figure S7: Sketch of the models, population and spin polarization evolution for (a) a one-bridge-site system, (b) a two-bridge-site system without spin-spin coupling on the second site, (c) a two-site-bridge system with spin-spin coupling on the second site. The model parameters are reported in Table II. $\Gamma = 4.3 \times 10^{-3}$ meV was set for all simulations.

Table II: Parameters for the simulations in Fig. S7 and Fig. S8. J, J_D, and D_z are taken from Table I.

	**Model a**	**Model b**	**Model c**
$J^{(0)}$	J	J	J
$J_D^{(0)}$	J_D	J_D	J_D
$D_z^{(0)}$	D_z	D_z	D_z
$J^{(1)}$	-	0	$J/4$
$J_D^{(1)}$	-	0	$J_D/4$
$D_z^{(1)}$	-	0	$D_z/3$
t_{01} (meV)	-	0.01	0.01

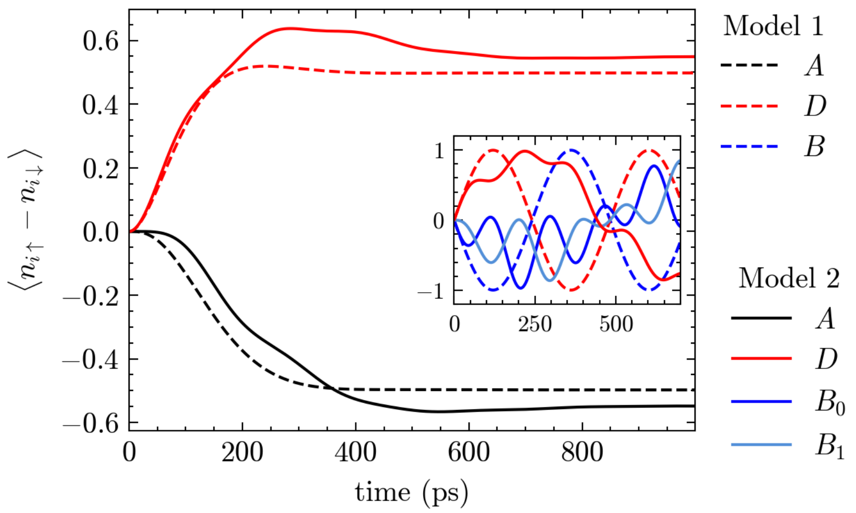

Figure S8: Main plot: simulated time evolution of the spin polarization on D and A for a system with one site on the bridge (dashed, parameters from model a in Table II) and for a system with two sites on the bridge (continuous, parameters from model b in Table II). Inset: coherent oscillations of the spin polarization on the donor bridge site(s). The population and local spin polarization evolution for each site are displayed in Fig. S7a and Fig. S7b.

on the donor, due to the same mechanism described in the main text, and there is no spin-coupling between electrons on the donor and on the acceptor. Since we are describing photoinduced electron transfer, we assume that the system starts in a singlet state. For the sake of simplicity, we assume in this derivation that the leading term in the spin-couplings is the DMI, that induces oscillations of the spin-polarization between -1 and +1, with angular frequency ω_i, as assumed in (S28), and verified numerically (see inset of Fig. S7 for the 1-bridge-site model).

An electron dwelling for a time t on a single site accumulates spin-polarization according to (S28). In the case of the multi-site model described above, the phases accumulated on each site i (given by $\omega_i t_i$) are additive. For dwell times $(t_0, \ldots, t_n)$, the total polarization is:

$$P(t_0, \ldots, t_n) = \sin\left(\sum_{i=1}^{n} \omega_i t_i\right) \tag{S43}$$

As the process is incoherent and Markovian, the dwell time t_i on site B_i follows an exponential distribution, described by the probability density:

$$p_i(t_i) = \Gamma_i e^{-\Gamma_i t_i} \tag{S44}$$

Assuming non-reversible incoherent transfer, the joint probability density is:

$$p(t_0, \ldots, t_n) = \prod_{i=1}^{n} \Gamma_i e^{-\Gamma_i t_i} \tag{S45}$$

The final polarization P_A is the average of $P(t_0, \ldots, t_n)$ over all possible dwell times:

$$P_A = \int_0^\infty \cdots \int_0^\infty \left[\prod_{i=1}^{n} \Gamma_i e^{-\Gamma_i t_i}\right] \sin\left(\sum_{i=1}^{n} \omega_i t_i\right) dt_0 \cdots dt_n \tag{S46}$$

Using the identity $\sin x = \mathrm{Im}(e^{ix})$, and combining the exponentials, we can factorize the integral

$$P_A = \mathrm{Im} \prod_{i=1}^{n} \left[\int_0^\infty \Gamma_i e^{-(\Gamma_i - i\omega_i)t_i} dt_i\right] \tag{S47}$$

The one-site integral evaluates to:

$$\int_0^\infty \Gamma_i e^{-(\Gamma_i - i\omega_i)t} dt = \frac{\Gamma_i}{\Gamma_i - i\omega_i} \tag{S48}$$

The compact exact formula for the polarization is therefore:

$$P_A = \mathrm{Im} \prod_{i=1}^{n} \frac{\Gamma_i}{\Gamma_i - i\omega_i} \tag{S49}$$

Notice that in the case of a single site (S49) collapses to (S34), thus recovering the 50% limit to spin polarization for systems with one bridge site.

To make the algebra easier in the case of more than one site, we adopt a polar form representation. Let $\theta_i = \arctan\left(\frac{\omega_i}{\Gamma_i}\right)$. We can rewrite the factor:

$$\frac{\Gamma_i}{\Gamma_i - i\omega_i} = \frac{\Gamma_i(\Gamma_i + i\omega_i)}{\Gamma_i^2 + \omega_i^2} = \cos^2\theta_i + i\sin\theta_i\cos\theta_i = \cos\theta_i e^{i\theta_i} \tag{S50}$$

Substituting into (S49) we obtain

$$P_A = \sin\left(\sum_{i=1}^{n} \theta_i\right) \prod_{i=1}^{n} \cos\theta_i, \quad \theta_i = \arctan\left(\frac{\omega_i}{\Gamma_i}\right) \tag{S51}$$

Now that we have an analytical form of the polarization we perform an optimization to maximize P_A. Specifically, we take the logarithmic derivative of $f(\theta_1, \ldots, \theta_n) = \sin(\Theta)\prod\cos\theta_i$ (where $\Theta = \sum\theta_i$):

$$\frac{\partial}{\partial\theta_j} \ln f = \cot\Theta - \tan\theta_j = 0$$

This implies all angles are equal: $\theta_1 = \theta_2 = \cdots = \theta_n = \theta$. In other words, the ratios between the frequency of oscillation of spin polarization on site B_i, ω_i and the rate Γ_i for transfer from site B_i to site B_{i+1} must be the same for all sites to maximize spin polarization.

The maximum polarization that can be obtained from a system with n bridge sites is obtained when $\tan\theta = \cot(n\theta)$ is true, which yields:

$$\theta = \frac{\pi}{2(n+1)} \tag{S52}$$

For a set of oscillation frequencies ω_i, imposed by the spin-couplings, the maximum polarization is achieved when the transfer rates are

$$\Gamma_i = \omega_i \cot\left(\frac{\pi}{2(n+1)}\right) \tag{S53}$$

The maximum polarization for n sites is:

$$P^{(n)}_{A,\max} = \cos^{n+1}\left(\frac{\pi}{2(n+1)}\right) \tag{S54}$$

Thus in the large-n limit, unitary spin polarization can be achieved.

Numerical simulations for systems with one, two, and three sites on the bridge are displayed in Fig. 4 and Fig. S9. The spin-coupling parameters are chosen so that the DMI is the leading term in the spin Hamiltonians (approximately twice the isotropic exchange) and the incoherent transfer rates are set to respect (S53) (the spin-coupling parameters and rates are reported in Table III). Specifically, we obtain $\approx 50\%$, $\approx 65\%$, and $\approx 73\%$ spin polarization for systems with one, two, or three sites on the bridge respectively, in agreement with (S54).

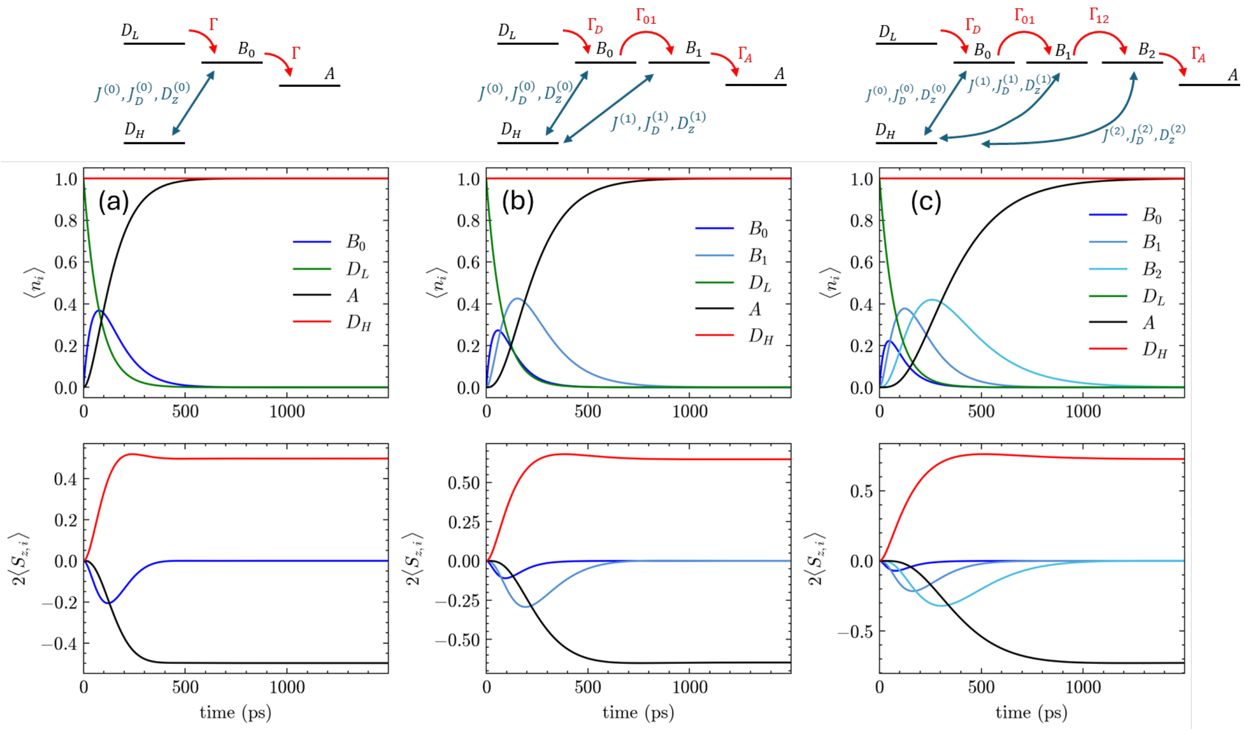

Figure S9: Sketch of the model, population and spin polarization evolution for systems with (a) one, (b) two and (c) three sites on the bridge, spin-spin couplings on each site and incoherent transfer rates are reported in Table III.

VI. AB-INITIO CALCULATIONS

Parameters $U, \Delta, t,$ and λ employed in the model presented in the main text were derived from ab-initio calculation on the PXX-NMI$_2$-NDI molecule [5]. Specifically, the on-site repulsion $U = 3.5\,\text{eV}$ was adopted from our previous study on the same system [6], where it was determined via CAS-sr-DFT calculations. Also the $\Delta = 5\,\text{eV}$ parameter is extracted from the same work. This value corresponds to the energy difference of the frontier molecular orbitals between the LUMO of the first site of the bridge (the NMI fragment closer to the PXX) and the HOMO localized on the donor at the LC-ωPBE\6-31G(d) level of theory, with a range separation parameter $\omega = 0.196 a_0^{-1}$. The hopping term t was obtained by performing DFT calculations on the PXX-NMI$_2$-NDI molecule at the LC-ωPBE\6-31G(d) and exploiting the Frontier Molecular Orbital (FMO) approach within the QChem software [7], to obtain the electronic coupling between the highest occupied orbital localized on the PXX donor and the lowest unoccupied on the first site of the NMI$_2$ bridge. In the FMO

Table III: Parameters for the simulations in Fig. S9 and Fig. 4. J, J_D, and D_z are taken from Table I. The rates are reported in meV units.

	**One-site**	**Two-site**	**Three-site**
$J^{(0)}$	J	J	J
$J_D^{(0)}$	J_D	J_D	J_D
$D_z^{(0)}$	D_z	D_z	D_z
$J^{(1)}$	-	$J/4$	$J/4$
$J_D^{(1)}$	-	$J_D/4$	$J_D/4$
$D_z^{(1)}$	-	$D_z/3$	$D_z/3$
$J^{(2)}$	-	-	$J/8$
$J_D^{(2)}$	-	-	$J_D/8$
$D_z^{(2)}$	-	-	$D_z/6$
Γ_D	4.3×10^{-3}	4.3×10^{-3}	4.3×10^{-3}
Γ_A	4.3×10^{-3}	2.48×10^{-3}	1.73×10^{-3}
Γ_{01}	-	7.45×10^{-3}	1.04×10^{-2}
Γ_{12}	-	-	3.46×10^{-3}

approach, the electronic coupling is defined as the off-diagonal matrix element of the Kohn-Sham operator between the a donor and an acceptor system. The molecule under study was divided into the donor part, comprising the PXX and the phenyl ring connecting the PXX and the NMI_2, and the acceptor one, including the rest of the molecule (NMI_2-NDI). The hopping term was, hence, given by:

$$t = \langle \phi_D^{HOMO} | \hat{f} | \phi_A^{NMI} \rangle = 1\,\text{meV} \tag{S55}$$

with $\hat{f}$ being the Kohn–Sham operator of the donor-acceptor system, ϕ_D^{HOMO} is the HOMO localized on the donor and ϕ_A^{NMI} the LUMO localized on the first site of the bridge. Finally, the spin-orbit coupling parameter $\lambda = 0.1\,\text{meV}$ was determined from the Cartesian components (λ_x, λ_y, λ_z) obtained via DFT calculations in ORCA [8] at the B3LYP\def2-TZVP level, using the relation $\lambda = \sqrt{\lambda_x^2 + \lambda_y^2 + \lambda_z^2}$.

VII. DERIVATION OF VIBRATIONALLY-MEDIATED INTERACTIONS IN A MULTI-ELECTRON CHIRAL BRIDGE

In this section we show that the same mechanism discussed in the minimal donor–bridge model also arises in a many-electron description of a chiral bridge. We consider a tight-binding chain in which low-energy Peierls modes modulate both the spin-independent hopping and the spin-orbit coupling. For clarity, we discuss the minimal case of two molecular sites coupled to a single vibrational mode. The generalization to a longer bridge and to several modes is straightforward.

We start from the Hamiltonian

$$H = \sum_{j=1}^{2} \sum_{\sigma=\uparrow,\downarrow} \epsilon_j c^\dagger_{j\sigma} c_{j\sigma} + \hbar\omega_0 \left(a^\dagger a + \frac{1}{2}\right) + \sum_{\sigma=\uparrow,\downarrow} c^\dagger_{1\sigma} c_{2\sigma} \left[-t - \frac{t_1}{\sqrt{2}}(a^\dagger + a)\right] + i\left[\lambda + \frac{\lambda_1}{\sqrt{2}}(a^\dagger + a)\right] \sum_{\sigma,\sigma'=\uparrow,\downarrow} c^\dagger_{1\sigma}(\sigma_z)_{\sigma\sigma'} c_{2\sigma'} + \text{h.c.}. \tag{S56}$$

Here t and λ are the static hopping and spin-orbit coupling, while t_1 and λ_1 are the corresponding Peierls coupling constants. The factor $1/\sqrt{2}$ is included in the definition of the dimensionless oscillator displacement.

Introducing the spinor

$$\mathbf{c} = \begin{pmatrix} c_{1\uparrow} \\ c_{1\downarrow} \\ c_{2\uparrow} \\ c_{2\downarrow} \end{pmatrix}, \qquad \mathbf{c}^\dagger = \begin{pmatrix} c^\dagger_{1\uparrow} & c^\dagger_{1\downarrow} & c^\dagger_{2\uparrow} & c^\dagger_{2\downarrow} \end{pmatrix}, \tag{S57}$$

the Hamiltonian can be written as

$$H = \mathbf{c}^\dagger H_f \mathbf{c} + \hbar\omega_0 \left(a^\dagger a + \frac{1}{2}\right) + \mathbf{c}^\dagger H_{\text{int}} \mathbf{c}(a^\dagger + a), \tag{S58}$$

where

$$H_f = \begin{pmatrix} \epsilon_1 & 0 & -t + i\lambda & 0 \\ 0 & \epsilon_1 & 0 & -t - i\lambda \\ -t - i\lambda & 0 & \epsilon_2 & 0 \\ 0 & -t + i\lambda & 0 & \epsilon_2 \end{pmatrix}, \tag{S59}$$

and

$$H_{\text{int}} = \frac{1}{\sqrt{2}} \begin{pmatrix} 0 & 0 & -t_1 + i\lambda_1 & 0 \\ 0 & 0 & 0 & -t_1 - i\lambda_1 \\ -t_1 - i\lambda_1 & 0 & 0 & 0 \\ 0 & -t_1 + i\lambda_1 & 0 & 0 \end{pmatrix}. \tag{S60}$$

Both H_f and H_{int} are Hermitian matrices. We now diagonalize the electron-vibration coupling matrix,

$$H_{\text{int}} = PQP^\dagger, \tag{S61}$$

where P is unitary and

$$Q = \frac{1}{\sqrt{2}} \begin{pmatrix} -\sqrt{t_1^2 + \lambda_1^2} & 0 & 0 & 0 \\ 0 & -\sqrt{t_1^2 + \lambda_1^2} & 0 & 0 \\ 0 & 0 & +\sqrt{t_1^2 + \lambda_1^2} & 0 \\ 0 & 0 & 0 & +\sqrt{t_1^2 + \lambda_1^2} \end{pmatrix}. \tag{S62}$$

A possible choice for P is

$$P = \frac{1}{\sqrt{2}} \begin{pmatrix} 0 & \frac{t_1 - i\lambda_1}{\sqrt{t_1^2 + \lambda_1^2}} & 0 & \frac{-t_1 + i\lambda_1}{\sqrt{t_1^2 + \lambda_1^2}} \\ \frac{t_1 + i\lambda_1}{\sqrt{t_1^2 + \lambda_1^2}} & 0 & \frac{-t_1 - i\lambda_1}{\sqrt{t_1^2 + \lambda_1^2}} & 0 \\ 0 & 1 & 0 & 1 \\ 1 & 0 & 1 & 0 \end{pmatrix}. \tag{S63}$$

We then define new fermionic operators

$$\mathbf{d} = P^\dagger \mathbf{c}, \qquad \mathbf{d}^\dagger = \mathbf{c}^\dagger P. \tag{S64}$$

Since P is unitary, the d_α operators obey the same fermionic anticommutation relations as the original $c_{j\sigma}$ operators. In this basis, the Hamiltonian becomes

$$\begin{aligned} H &= \mathbf{d}^\dagger \widetilde{H}_f \mathbf{d} + \hbar\omega_0 \left(a^\dagger a + \frac{1}{2} \right) + \mathbf{d}^\dagger Q \mathbf{d} (a^\dagger + a) \\ &= \sum_{\alpha,\beta=1}^{4} (\widetilde{H}_f)_{\alpha\beta} d_\alpha^\dagger d_\beta + \hbar\omega_0 \left(a^\dagger a + \frac{1}{2} \right) + \sum_{\alpha=1}^{4} q_\alpha d_\alpha^\dagger d_\alpha (a^\dagger + a), \end{aligned} \tag{S65}$$

with

$$\widetilde{H}_f = P^\dagger H_f P. \tag{S66}$$

The indices α, β label the eigenmodes of the electron-vibration coupling matrix, while $j = 1, 2$ labels the original molecular sites. We observe that in the new basis the transformed fermionic matrix

$$\hat{H}_f = P^\dagger H_f P \tag{S67}$$

keeps the same spin structure as the original one. Hence, it can be written in the form

$$\hat{H}_f = \begin{pmatrix} \tilde{\epsilon}_1 & 0 & -\tilde{t} + i\tilde{\lambda} & 0 \\ 0 & \tilde{\epsilon}_1 & 0 & -\tilde{t} - i\tilde{\lambda} \\ -\tilde{t} - i\tilde{\lambda} & 0 & \tilde{\epsilon}_2 & 0 \\ 0 & -\tilde{t} + i\tilde{\lambda} & 0 & \tilde{\epsilon}_2 \end{pmatrix}, \tag{S68}$$

where the effective parameters $\tilde{\epsilon}_j$, $\tilde{t}$ and $\tilde{\lambda}$ depend on the microscopic parameters and on the matrix P.

Since the spin structure is preserved, we can rewrite the fermionic part in tight-binding form as

$$\begin{aligned} \mathbf{d}^\dagger \hat{H}_f \mathbf{d} = & \sum_{j=1}^{2} \sum_{\sigma=\uparrow,\downarrow} \tilde{\epsilon}_j d^\dagger_{j,\sigma} d_{j,\sigma} \\ & + \left[\sum_{\sigma,\sigma'=\uparrow,\downarrow} d^\dagger_{1,\sigma} \left(-\tilde{t}\, \mathbf{1}_{2\times 2} + i\tilde{\lambda}\sigma_z \right)_{\sigma\sigma'} d_{2,\sigma'} + \text{h.c.} \right]. \end{aligned} \tag{S69}$$

Therefore, after diagonalizing the electron-vibration coupling matrix, the Hamiltonian reads

$$\begin{aligned} H = & \sum_{j=1}^{2} \sum_{\sigma=\uparrow,\downarrow} \tilde{\epsilon}_j d^\dagger_{j,\sigma} d_{j,\sigma} \\ & + \left[\sum_{\sigma,\sigma'=\uparrow,\downarrow} d^\dagger_{1,\sigma} \left(-\tilde{t}\, \mathbf{1}_{2\times 2} + i\tilde{\lambda}\sigma_z \right)_{\sigma\sigma'} d_{2,\sigma'} + \text{h.c.} \right] \\ & + \hbar\omega_0 \left(a^\dagger a + \frac{1}{2} \right) + \sum_{j=1}^{2} \sum_{\sigma=\uparrow,\downarrow} q_{j,\sigma} d^\dagger_{j,\sigma} d_{j,\sigma} (a^\dagger + a). \end{aligned} \tag{S70}$$

For the present model the eigenvalues of Q are spin-degenerate, so that

$$q_{j,\uparrow} = q_{j,\downarrow} \equiv q_j, \tag{S71}$$

with

$$q_1 = -\frac{\sqrt{t_1^2 + \lambda_1^2}}{\sqrt{2}}, \qquad q_2 = +\frac{\sqrt{t_1^2 + \lambda_1^2}}{\sqrt{2}}. \tag{S72}$$

We now apply a Lang-Firsov transformation,

$$\tilde{H} = e^{S} H e^{-S}, \tag{S73}$$

with

$$S = \sum_{j=1}^{2} \sum_{\sigma=\uparrow,\downarrow} \frac{q_{j,\sigma}}{\hbar\omega_0} d_{j,\sigma}^{\dagger} d_{j,\sigma} (a^{\dagger} - a). \tag{S74}$$

Since $S^{\dagger} = -S$, this transformation is unitary. The transformed operators are

$$e^{S} a e^{-S} = a - \sum_{j=1}^{2} \sum_{\sigma=\uparrow,\downarrow} \frac{q_{j,\sigma}}{\hbar\omega_0} d_{j,\sigma}^{\dagger} d_{j,\sigma}, \tag{S75}$$

$$e^{S} a^{\dagger} e^{-S} = a^{\dagger} - \sum_{j=1}^{2} \sum_{\sigma=\uparrow,\downarrow} \frac{q_{j,\sigma}}{\hbar\omega_0} d_{j,\sigma}^{\dagger} d_{j,\sigma}, \tag{S76}$$

and

$$e^{S} d_{j,\sigma} e^{-S} = d_{j,\sigma} \exp\left[-\frac{q_{j,\sigma}}{\hbar\omega_0} (a^{\dagger} - a) \right], \tag{S77}$$

$$e^{S} d_{j,\sigma}^{\dagger} e^{-S} = d_{j,\sigma}^{\dagger} \exp\left[+\frac{q_{j,\sigma}}{\hbar\omega_0} (a^{\dagger} - a) \right]. \tag{S78}$$

The density operators are unchanged,

$$e^{S} d_{j,\sigma}^{\dagger} d_{j,\sigma} e^{-S} = d_{j,\sigma}^{\dagger} d_{j,\sigma} \equiv n_{j,\sigma}^{d}. \tag{S79}$$

The hopping terms instead acquire displacement operators:

$$e^{S} d_{1,\sigma}^{\dagger} d_{2,\sigma'} e^{-S} = d_{1,\sigma}^{\dagger} d_{2,\sigma'} \exp\left[\frac{q_{1,\sigma} - q_{2,\sigma'}}{\hbar\omega_0} (a^{\dagger} - a) \right]. \tag{S80}$$

Substituting these expressions into the Hamiltonian, the linear electron-vibration coupling cancels exactly. The local part becomes

$$\begin{aligned} &\sum_{j=1}^{2} \sum_{\sigma} \tilde{\epsilon}_j n_{j,\sigma}^{d} + \hbar\omega_0 \left(\tilde{a}^{\dagger} \tilde{a} + \frac{1}{2} \right) + \sum_{j=1}^{2} \sum_{\sigma} q_{j,\sigma} n_{j,\sigma}^{d} (\tilde{a}^{\dagger} + \tilde{a}) \\ &= \sum_{j=1}^{2} \sum_{\sigma} \tilde{\epsilon}_j n_{j,\sigma}^{d} + \hbar\omega_0 \left(a^{\dagger} a + \frac{1}{2} \right) - \frac{1}{\hbar\omega_0} \left[\sum_{j=1}^{2} \sum_{\sigma} q_{j,\sigma} n_{j,\sigma}^{d} \right]^2 . \end{aligned} \tag{S81}$$

Using $q_{j,\uparrow} = q_{j,\downarrow} \equiv q_j$ and the fermionic identity

$$\left(n_{j,\sigma}^{d} \right)^2 = n_{j,\sigma}^{d}, \tag{S82}$$

we obtain

$$\left[\sum_{j=1}^{2}\sum_{\sigma} q_{j,\sigma} n^d_{j,\sigma}\right]^2 = q_1^2\left(n^d_{1,\uparrow} + n^d_{1,\downarrow} + 2n^d_{1,\uparrow}n^d_{1,\downarrow}\right) + q_2^2\left(n^d_{2,\uparrow} + n^d_{2,\downarrow} + 2n^d_{2,\uparrow}n^d_{2,\downarrow}\right) + 2q_1q_2\left(n^d_{1,\uparrow}n^d_{2,\uparrow} + n^d_{1,\uparrow}n^d_{2,\downarrow} + n^d_{1,\downarrow}n^d_{2,\uparrow} + n^d_{1,\downarrow}n^d_{2,\downarrow}\right). \tag{S83}$$

Hence the transformed Hamiltonian is

$$\begin{aligned}\tilde{H} = &\sum_{j=1}^{2}\sum_{\sigma=\uparrow,\downarrow}\left(\tilde{\epsilon}_j - \frac{q_j^2}{\hbar\omega_0}\right)n^d_{j,\sigma} + \hbar\omega_0\left(a^\dagger a + \frac{1}{2}\right)\\ &+ \left[\sum_{\sigma,\sigma'} d^\dagger_{1,\sigma}\left(-\tilde{t}\,\mathbf{1}_{2\times 2} + i\tilde{\lambda}\sigma_z\right)_{\sigma\sigma'} d_{2,\sigma'}\exp\left[\frac{q_1-q_2}{\hbar\omega_0}(a^\dagger - a)\right] + \text{h.c.}\right]\\ &- \sum_{j=1}^{2}\frac{2q_j^2}{\hbar\omega_0}n^d_{j,\uparrow}n^d_{j,\downarrow}\\ &- \frac{2q_1q_2}{\hbar\omega_0}\left(n^d_{1,\uparrow}n^d_{2,\uparrow} + n^d_{1,\uparrow}n^d_{2,\downarrow} + n^d_{1,\downarrow}n^d_{2,\uparrow} + n^d_{1,\downarrow}n^d_{2,\downarrow}\right).\end{aligned} \tag{S84}$$

We now rewrite the phonon-mediated density-density interaction in the original site basis. Since

$$\sum_{j=1}^{2}\sum_{\sigma=\uparrow,\downarrow} q_{j,\sigma}n^d_{j,\sigma} = \mathbf{c}^\dagger H_{\text{int}}\mathbf{c}, \tag{S85}$$

the interaction generated by the Lang-Firsov transformation can be equivalently written as

$$H_{\text{ph-med}} = -\frac{1}{\hbar\omega_0}\left(\mathbf{c}^\dagger H_{\text{int}}\mathbf{c}\right)^2. \tag{S86}$$

Using the explicit form of H_{int}, this quartic term can be decomposed into charge and spin channels. Apart from pair-hopping terms, which do not contribute in the singly-occupied low-energy spin sector considered here, one obtains

$$H_{\text{ph-med}} = \frac{1}{\hbar\omega_0}\left[t_1^2 H_{\text{H}} + \lambda_1^2 H_{\text{XXZ}} - 4t_1\lambda_1 H_{\text{DM}}\right] + H_{\text{pair}}, \tag{S87}$$

where

$$H_{\text{pair}} = -\frac{t_1^2+\lambda_1^2}{\hbar\omega_0}\left(c^\dagger_{1\downarrow}c_{2\downarrow}c^\dagger_{1\uparrow}c_{2\uparrow} + c^\dagger_{2\downarrow}c_{1\downarrow}c^\dagger_{2\uparrow}c_{1\uparrow}\right) \tag{S88}$$

is a pair-hopping contribution. The spin operators entering Eq. (S87) are

$$H_{\text{H}} = 2S_1^zS_2^z + \frac{1}{2}n_1n_2 + S_1^-S_2^+ + S_1^+S_2^-, \tag{S89}$$

$$H_{\rm XXZ} = 2S_1^zS_2^z + \frac{1}{2}n_1n_2 - \left(S_1^-S_2^+ + S_1^+S_2^-\right), \tag{S90}$$

and

$$H_{\rm DM} = \left(\mathbf{S}_1 \times \mathbf{S}_2\right)_z = S_1^xS_2^y - S_1^yS_2^x. \tag{S91}$$

Here

$$S_j^+ = c_{j\uparrow}^\dagger c_{j\downarrow}, \qquad S_j^- = c_{j\downarrow}^\dagger c_{j\uparrow}, \qquad S_j^z = \frac{1}{2}\left(n_{j\uparrow} - n_{j\downarrow}\right), \tag{S92}$$

and $n_j = n_{j\uparrow} + n_{j\downarrow}$.

To obtain Eq. (S87), we used the identities

$$c_{1\downarrow}^\dagger c_{1\uparrow} c_{2\uparrow}^\dagger c_{2\downarrow} = S_1^-S_2^+, \qquad c_{1\uparrow}^\dagger c_{1\downarrow} c_{2\downarrow}^\dagger c_{2\uparrow} = S_1^+S_2^-, \tag{S93}$$

$$n_{1\downarrow}n_{2\downarrow} + n_{1\uparrow}n_{2\uparrow} = 2S_1^zS_2^z + \frac{1}{2}n_1n_2, \tag{S94}$$

and

$$i\left(S_1^-S_2^+ - S_1^+S_2^-\right) = -2\left(\mathbf{S}_1 \times \mathbf{S}_2\right)_z. \tag{S95}$$

In the low-energy subspace with one electron on each site, $n_1 = n_2 = 1$ and the pair-hopping term $H_{\rm pair}$ connects the singly-occupied sector to doubly occupied configurations. Therefore, it can either be neglected in the projected spin Hamiltonian or treated perturbatively as an additional high-energy correction. Dropping constant charge terms, Eq. (S87) reduces to an effective spin interaction of the form

$$H_{\rm spin}^{\rm ph} = J_{\rm ph}\mathbf{S}_1 \cdot \mathbf{S}_2 + J_{\rm ph}^D\left(2S_1^zS_2^z - S_1^xS_2^x - S_1^yS_2^y\right) + D_{\rm ph}^z\left(S_1^xS_2^y - S_1^yS_2^x\right), \tag{S96}$$

with couplings proportional to

$$J_{\rm ph} \propto \frac{t_1^2}{\hbar\omega_0}, \qquad J_{\rm ph}^D \propto \frac{\lambda_1^2}{\hbar\omega_0}, \qquad D_{\rm ph}^z \propto -\frac{4t_1\lambda_1}{\hbar\omega_0}. \tag{S97}$$

Hence, Peierls vibrations modulating hopping and spin-orbit coupling generate respectively an isotropic exchange, an axial anisotropic exchange, and a Dzyaloshinskii-Moriya interaction. In particular, the antisymmetric exchange is linear in both t_1 and λ_1, showing that it directly originates from the interference between the vibrational modulation of hopping and that of spin-orbit coupling.

A. Residual electron-vibration terms

For completeness, we also report the structure of the remaining terms generated by the Lang-Firsov transformation. These terms are less relevant for the discussion of the effective spin-spin interaction, but they show explicitly how the single-particle part of the Hamiltonian is dressed by the vibrational displacement. We first introduce

$$X = a^\dagger - a, \qquad D_{12} = e^{\alpha_{12} X}, \qquad \alpha_{12} = \frac{q_1 - q_2}{\hbar\omega_0} = -\frac{\sqrt{2(t_1^2 + \lambda_1^2)}}{\hbar\omega_0}, \tag{S98}$$

where $q_1 = -\sqrt{t_1^2 + \lambda_1^2}/\sqrt{2}$ and $q_2 = +\sqrt{t_1^2 + \lambda_1^2}/\sqrt{2}$. We also define the symmetric and antisymmetric hopping operators

$$h_\sigma^+ = c_{1\sigma}^\dagger c_{2\sigma} + c_{2\sigma}^\dagger c_{1\sigma}, \qquad h_\sigma^- = c_{1\sigma}^\dagger c_{2\sigma} - c_{2\sigma}^\dagger c_{1\sigma}. \tag{S99}$$

Transforming back from the d basis to the original c basis, the one-body part of the Hamiltonian can be written as the sum of a static renormalized contribution and a displacement-dependent contribution,

$$\widetilde{H}_{\text{1b}} = \widetilde{H}_{\text{1b}}^{(0)} + \widetilde{H}_{\text{1b}}^{(\text{vib})}. \tag{S100}$$

The static term is

$$\begin{aligned} \widetilde{H}_{\text{1b}}^{(0)} = & \left[\frac{\epsilon_1 + \epsilon_2}{2} - \frac{t_1^2 + \lambda_1^2}{2\hbar\omega_0}\right] \sum_{j=1}^{2} \sum_{\sigma} c_{j\sigma}^\dagger c_{j\sigma} \\ & - \frac{t_1 t + \lambda_1 \lambda}{t_1^2 + \lambda_1^2} \left[t_1 \sum_\sigma \left(c_{1\sigma}^\dagger c_{2\sigma} + c_{2\sigma}^\dagger c_{1\sigma} \right) - \lambda_1 \sum_{\sigma\sigma'} \left(i c_{1\sigma}^\dagger (\sigma_z)_{\sigma\sigma'} c_{2\sigma'} + \text{h.c.} \right) \right]. \end{aligned} \tag{S101}$$

The first line is a uniform polaronic energy shift, while the second line corresponds to a renormalization of the static hopping and spin-orbit amplitudes.

The displacement-dependent part reads

$$\begin{aligned} \widetilde{H}_{\text{1b}}^{(\text{vib})} = & \frac{\epsilon_1 - \epsilon_2}{2} \cosh(\alpha_{12} X) \sum_\sigma (n_{1\sigma} - n_{2\sigma}) \\ & - i \frac{t_1 \lambda - \lambda_1 t}{\sqrt{t_1^2 + \lambda_1^2}} \sinh(\alpha_{12} X)\, 2(S_1^z - S_2^z) \\ & - \frac{t_1}{\sqrt{t_1^2 + \lambda_1^2}} \frac{\epsilon_1 - \epsilon_2}{2} \sinh(\alpha_{12} X) \left(h_\downarrow^- + h_\uparrow^- \right) \\ & - \frac{i\lambda_1}{\sqrt{t_1^2 + \lambda_1^2}} \frac{\epsilon_1 - \epsilon_2}{2} \sinh(\alpha_{12} X) \left(h_\downarrow^+ - h_\uparrow^+ \right) \\ & + i \frac{t_1 (t_1 \lambda - \lambda_1 t)}{t_1^2 + \lambda_1^2} \cosh(\alpha_{12} X) \left(-h_\downarrow^- + h_\uparrow^- \right) \\ & + \frac{\lambda_1 (t_1 \lambda - \lambda_1 t)}{t_1^2 + \lambda_1^2} \cosh(\alpha_{12} X) \left(h_\downarrow^+ + h_\uparrow^+ \right). \end{aligned} \tag{S102}$$

These terms describe phonon-dressed single-particle processes. In particular, they include displacement-dependent site-energy imbalance, spin-dependent on-site terms proportional to $S_1^z - S_2^z$, and phonon-dressed hopping and spin-orbit hopping operators.

Combining the one-body terms with the phonon-mediated two-body interaction discussed above, the transformed Hamiltonian can be written as

$$\widetilde{H} = \widetilde{H}_{1b}^{(0)} + \widetilde{H}_{1b}^{(\mathrm{vib})} + \hbar\omega_0 \left(a^\dagger a + \frac{1}{2} \right) + H_{\mathrm{ph-med}}. \tag{S103}$$

Here the last term is the effective phonon-mediated interaction,

$$H_{\mathrm{ph-med}} = \frac{1}{\hbar\omega_0} \left[t_1^2 H_{\mathrm{H}} + \lambda_1^2 H_{\mathrm{XXZ}} - 4t_1\lambda_1 H_{\mathrm{DM}} \right] + H_{\mathrm{pair}}, \tag{S104}$$

with

$$H_{\mathrm{pair}} = -\frac{t_1^2 + \lambda_1^2}{\hbar\omega_0} \left(c_{1\downarrow}^\dagger c_{2\downarrow} c_{1\uparrow}^\dagger c_{2\uparrow} + c_{2\downarrow}^\dagger c_{1\downarrow} c_{2\uparrow}^\dagger c_{1\uparrow} \right). \tag{S105}$$

The operators H_{H}, H_{XXZ} and H_{DM} are those defined in Eqs. (S89)–(S91). Therefore, while Eqs. (S101) and (S102) only renormalize and dress the single-particle sector, Eq. (S104) contains the central result: Peierls vibrations generate effective electron-electron interactions with isotropic, anisotropic and Dzyaloshinskii-Moriya spin structure.

VIII. VIBRATION DAMPING

We start from the two-site electronic Hamiltonian coupled to a single vibrational mode through Peierls-like modulations of both the hopping amplitude and the spin-orbit coupling. In the presence of a local Hubbard interaction, the Hamiltonian is written as

$$H = H_0 + H_{\mathrm{int}}, \tag{S106}$$

where

$$H_0 = \hbar\omega_0 \left(a^\dagger a + \frac{1}{2} \right) + \sum_{j=1}^{2} \sum_{\sigma=\uparrow,\downarrow} \epsilon_j n_{j\sigma} + U \sum_{j=1}^{2} n_{j\uparrow} n_{j\downarrow}, \tag{S107}$$

with

$$n_{j\sigma} = c_{j\sigma}^\dagger c_{j\sigma}. \tag{S108}$$

The electron-boson interaction is

$$H_{\mathrm{int}} = (a^\dagger + a) \sum_{\sigma=\uparrow,\downarrow} \left[(-t_1 + i\sigma\lambda_1) c_{1\sigma}^\dagger c_{2\sigma} + (-t_1 - i\sigma\lambda_1) c_{2\sigma}^\dagger c_{1\sigma} \right], \tag{S109}$$

where we use the convention

$$\sigma = +1 \quad \text{for} \quad \uparrow, \qquad \sigma = -1 \quad \text{for} \quad \downarrow . \tag{S110}$$

We now look for an anti-Hermitian generator S such that the electron-boson interaction is removed at first order in the unitary transformation

$$\widetilde{H} = e^{S} H e^{-S}. \tag{S111}$$

Expanding to first order gives

$$\widetilde{H} = H + [S, H] + \cdots . \tag{S112}$$

Therefore, the condition for cancelling H_{int} at first order is

$$[S, H_0] + H_{\text{int}} = 0. \tag{S113}$$

Differently from the standard Lang-Firsov case, the hopping operators appearing in H_{int} do not commute with the electronic part of H_0. Indeed, the energy denominators depend on whether the hopping process creates, destroys, or leaves unchanged a double occupation. We define

$$\Delta = \epsilon_1 - \epsilon_2. \tag{S114}$$

For the hopping process $2 \to 1$, we introduce the projectors

$$\begin{aligned} P^{0}_{21,\sigma} &= (1 - n_{1\bar{\sigma}})(1 - n_{2\bar{\sigma}}) + n_{1\bar{\sigma}} n_{2\bar{\sigma}}, \\ P^{+}_{21,\sigma} &= n_{1\bar{\sigma}}(1 - n_{2\bar{\sigma}}), \\ P^{-}_{21,\sigma} &= (1 - n_{1\bar{\sigma}}) n_{2\bar{\sigma}}. \end{aligned} \tag{S115}$$

Here $\bar{\sigma}$ denotes the spin opposite to σ. The three projectors select, respectively, processes that leave the number of double occupations unchanged, create one double occupation, or remove one double occupation.

Similarly, for the hopping process $1 \to 2$, we define

$$\begin{aligned} P^{0}_{12,\sigma} &= (1 - n_{2\bar{\sigma}})(1 - n_{1\bar{\sigma}}) + n_{2\bar{\sigma}} n_{1\bar{\sigma}}, \\ P^{+}_{12,\sigma} &= n_{2\bar{\sigma}}(1 - n_{1\bar{\sigma}}), \\ P^{-}_{12,\sigma} &= (1 - n_{2\bar{\sigma}}) n_{1\bar{\sigma}}. \end{aligned} \tag{S116}$$

The generator which solves

$$[S, H_0] + H_{\rm int} = 0 \tag{S117}$$

is

$$\begin{aligned} S = \sum_\sigma (-t_1 + i\sigma\lambda_1) \Bigg[& P^0_{21,\sigma} c^\dagger_{1\sigma} c_{2\sigma} \frac{\hbar\omega_0(a^\dagger - a) - \Delta(a^\dagger + a)}{(\hbar\omega_0)^2 - \Delta^2} \\ & + P^+_{21,\sigma} c^\dagger_{1\sigma} c_{2\sigma} \frac{\hbar\omega_0(a^\dagger - a) - (\Delta + U)(a^\dagger + a)}{(\hbar\omega_0)^2 - (\Delta + U)^2} \\ & + P^-_{21,\sigma} c^\dagger_{1\sigma} c_{2\sigma} \frac{\hbar\omega_0(a^\dagger - a) - (\Delta - U)(a^\dagger + a)}{(\hbar\omega_0)^2 - (\Delta - U)^2} \Bigg] \\ + \sum_\sigma (-t_1 - i\sigma\lambda_1) \Bigg[& P^0_{12,\sigma} c^\dagger_{2\sigma} c_{1\sigma} \frac{\hbar\omega_0(a^\dagger - a) + \Delta(a^\dagger + a)}{(\hbar\omega_0)^2 - \Delta^2} \\ & + P^+_{12,\sigma} c^\dagger_{2\sigma} c_{1\sigma} \frac{\hbar\omega_0(a^\dagger - a) - (-\Delta + U)(a^\dagger + a)}{(\hbar\omega_0)^2 - (-\Delta + U)^2} \\ & + P^-_{12,\sigma} c^\dagger_{2\sigma} c_{1\sigma} \frac{\hbar\omega_0(a^\dagger - a) - (-\Delta - U)(a^\dagger + a)}{(\hbar\omega_0)^2 - (-\Delta - U)^2} \Bigg]. \end{aligned} \tag{S118}$$

This expression was checked symbolically, using the `sneg` package, to satisfy

$$[S, H_0] + H_{\rm int} = 0. \tag{S119}$$

Therefore, the transformed Hamiltonian has no electron-boson coupling at first order:

$$\widetilde{H} = H_0 + \frac{1}{2}[S, H_{\rm int}] + \cdots . \tag{S120}$$

The crucial difference with respect to the ordinary Lang-Firsov transformation is that the generator is not controlled only by the phonon energy $\hbar\omega_0$. Instead, the denominators also contain the electronic excitation energies Δ, $\Delta + U$, $\Delta - U$, and their counterparts for the reverse hopping process.

A. Transformation of the bosonic annihilation operator

We now compute the transformation of the bosonic annihilation operator a. Under the same unitary transformation,

$$\widetilde{a} = e^S a e^{-S}, \tag{S121}$$

and, to first order in the generator S,

$$\widetilde{a} = a + [S, a] + \mathcal{O}(S^2). \tag{S122}$$

Since the fermionic operators commute with a, the commutator acts only on the bosonic part of the generator. We use

$$[a^\dagger - a, a] = -1, \qquad [a^\dagger + a, a] = -1. \tag{S123}$$

Therefore, for a generic term of the form

$$\mathcal{O}\frac{\hbar\omega_0(a^\dagger - a) - \delta(a^\dagger + a)}{(\hbar\omega_0)^2 - \delta^2}, \tag{S124}$$

where $\mathcal{O}$ is a purely fermionic operator, one obtains

$$\left[\mathcal{O}\frac{\hbar\omega_0(a^\dagger - a) - \delta(a^\dagger + a)}{(\hbar\omega_0)^2 - \delta^2}, a\right] = \mathcal{O}\frac{-\hbar\omega_0 + \delta}{(\hbar\omega_0)^2 - \delta^2} = -\frac{\mathcal{O}}{\hbar\omega_0 + \delta}. \tag{S125}$$

Applying this identity to all terms in S, we find

$$\begin{aligned}[S, a] = &-\sum_\sigma(-t_1 + i\sigma\lambda_1)\left[\frac{P^0_{21,\sigma}c^\dagger_{1\sigma}c_{2\sigma}}{\hbar\omega_0 + \Delta} + \frac{P^+_{21,\sigma}c^\dagger_{1\sigma}c_{2\sigma}}{\hbar\omega_0 + \Delta + U} + \frac{P^-_{21,\sigma}c^\dagger_{1\sigma}c_{2\sigma}}{\hbar\omega_0 + \Delta - U}\right] \\ &-\sum_\sigma(-t_1 - i\sigma\lambda_1)\left[\frac{P^0_{12,\sigma}c^\dagger_{2\sigma}c_{1\sigma}}{\hbar\omega_0 - \Delta} + \frac{P^+_{12,\sigma}c^\dagger_{2\sigma}c_{1\sigma}}{\hbar\omega_0 - \Delta + U} + \frac{P^-_{12,\sigma}c^\dagger_{2\sigma}c_{1\sigma}}{\hbar\omega_0 - \Delta - U}\right].\end{aligned} \tag{S126}$$

Equivalently, the transformed bosonic annihilation operator at first order is

$$\begin{aligned}\widetilde{a} = a &-\sum_\sigma(-t_1 + i\sigma\lambda_1)\left[\frac{P^0_{21,\sigma}c^\dagger_{1\sigma}c_{2\sigma}}{\hbar\omega_0 + \Delta} + \frac{P^+_{21,\sigma}c^\dagger_{1\sigma}c_{2\sigma}}{\hbar\omega_0 + \Delta + U} + \frac{P^-_{21,\sigma}c^\dagger_{1\sigma}c_{2\sigma}}{\hbar\omega_0 + \Delta - U}\right] \\ &-\sum_\sigma(-t_1 - i\sigma\lambda_1)\left[\frac{P^0_{12,\sigma}c^\dagger_{2\sigma}c_{1\sigma}}{\hbar\omega_0 - \Delta} + \frac{P^+_{12,\sigma}c^\dagger_{2\sigma}c_{1\sigma}}{\hbar\omega_0 - \Delta + U} + \frac{P^-_{12,\sigma}c^\dagger_{2\sigma}c_{1\sigma}}{\hbar\omega_0 - \Delta - U}\right] + \mathcal{O}(S^2).\end{aligned} \tag{S127}$$

This result shows that, in the presence of the Hubbard interaction, the bosonic displacement is not controlled by a density operator, as in the ordinary Lang-Firsov transformation. Instead, a is shifted by projected hopping operators. The different denominators correspond to virtual processes which either leave the number of double occupations unchanged, create a double occupation, or destroy a double occupation.

---

[1] D. Tupkary, A. Dhar, M. Kulkarni, and A. Purkayastha, Fundamental limitations in lindblad descriptions of systems weakly coupled to baths, Phys. Rev. A **105**, 032208 (2022).

[2] T. P. Fay, L. P. Lindoy, and D. E. Manolopoulos, Spin-selective electron transfer reactions of radical pairs: Beyond the haberkorn master equation, J. Chem. Phys. **149** (2018).

[3] A. Trushechkin, Quantum master equations and steady states for the ultrastrong-coupling limit and the strong-decoherence limit, Physical Review A **106**, 042209 (2022).

[4] Only if $lambda_0, t_0 \neq 0$ a small matrix element mixing singlet and triplet appears with a $t_1 \lambda 1/\Delta'$ prefactor appears.

[5] H. J. Eckvahl, N. A. Tcyrulnikov, A. Chiesa, J. M. Bradley, R. M. Young, S. Carretta, M. D. Krzyaniak, and M. R. Wasielewski, Direct observation of chirality-induced spin selectivity in electron donor–acceptor molecules, Science **382**, 197 (2023).

[6] D. K. A. Phan Huu, A. Cantarella, P. Bonfà, L. Savi, A. Chiesa, A. Painelli, and S. Carretta, Ab initio parametrization of a generalized Hubbard model in a molecule displaying chirality-induced spin selectivity, Commun. Mater. **6**, 1 (2025).

[7] E. Epifanovsky *et al.*, Software for the frontiers of quantum chemistry: An overview of developments in the q-chem 5 package, J. Chem. Phys. **155**, 084801 (2021).

[8] F. Neese, F. Wennmohs, U. Becker, and C. Riplinger, The orca quantum chemistry program package, J. Chem. Phys. **152**, 224108 (2020).
